# Myxoma Virus Expressing LIGHT (TNFSF14) Pre-Loaded into Adipose-Derived Mesenchymal Stem Cells Is Effective Treatment for Murine Pancreatic Adenocarcinoma

**DOI:** 10.3390/cancers13061394

**Published:** 2021-03-19

**Authors:** Joanna Jazowiecka-Rakus, Agata Hadrys, Masmudur M. Rahman, Grant McFadden, Wojciech Fidyk, Ewa Chmielik, Marlena Pazdzior, Maciej Grajek, Violetta Kozik, Aleksander Sochanik

**Affiliations:** 1 Center for Translational Research and Molecular Biology of Cancer, Maria Sklodowska-Curie National Research Institute of Oncology, Gliwice Branch, Wybrzeze AK 15, 44-102 Gliwice, Poland; Agata.Hadrys@io.gliwice.pl (A.H.); Marlena.Pazdzior@io.gliwice.pl (M.P.); Aleksander.Sochanik@io.gliwice.pl (A.S.); 2 Institute of Chemistry, University of Silesia, Szkolna 9, 40-007 Katowice, Poland; violetta.kozik@us.edu.pl; 3 Biodesign Institute, Arizona State University, Tempe, AZ 85287, USA; Masmudur.Rahman@asu.edu (M.M.R.); grantmcf@asu.edu (G.M.); 4 Department of Bone Marrow Transplantation and Hematology-Oncology, Maria Sklodowska-Curie National Research Institute of Oncology, Gliwice Branch, Wybrzeze AK 15, 44-102 Gliwice, Poland; Wojciech.Fidyk@io.gliwice.pl; 5 Tumor Pathology Department, Maria Sklodowska-Curie National Research Institute of Oncology, Gliwice Branch, Wybrzeze AK 15, 44-102 Gliwice, Poland; Ewa.Chmielik@io.gliwice.pl; 6 Oncological and Reconstructive Surgery Department, Maria Sklodowska-Curie National Research Institute of Oncology, Gliwice Branch, Wybrzeze AK 15, 44-102 Gliwice, Poland; Maciej.Grajek@io.gliwice.pl

**Keywords:** adipose tissue-derived stem cells (ADSCs), mesenchymal stem cells, oncolytic virus, myxoma virus, oncolytic virotherapy, pancreatic ductal adenocarcinoma

## Abstract

**Simple Summary:**

Pancreatic cancer is a deadly disease with no effective therapy. Oncolytic viruses such as myxoma (MYXV) have revealed great potential to treat malignancies, due to dual anti-cancer effects—oncolytic and immune-stimulating effects. We aimed to verify whether adipose-derived mesenchymal stem cells (ADSCs) pre-loaded ex vivo with transgene-armed myxoma construct would be useful for transferring the virus to murine pancreatic lesions and whether this would reduce tumor burden. We confirmed that the carrier cells remained viable after infection, in contrast to pancreatic cancer cells, which were destroyed. Intraperitoneal (IP) administration of the shielded virus (ADSCs/MYXV) revealed localization in the pancreas, decreased tumor burden and an adaptive anti-tumor immune response. We conclude that ADSCs pre-loaded with recombinant MYXV and administered IP allowed for the ferrying of the virus to pancreatic cancer lesions, followed by tumor regression and extended survival in the treated mice. This therapeutic approach has excellent potential for treating pancreatic cancer.

**Abstract:**

Pancreatic ductal adenocarcinoma (PDAC) is a weakly immunogenic fatal neoplasm. Oncolytic viruses with dual anti-cancer properties—oncolytic and immune response-boosting effects—have great potential for PDAC management. Adipose-derived stem cells (ADSCs) of mesenchymal origin were infected ex vivo with recombinant myxoma virus (MYXV), which encodes murine LIGHT, also called tumor necrosis factor ligand superfamily member 14 (TNFSF14). The viability and proliferation of ADSCs were not remarkably decreased (1–2 days) following MYXV infection, in sharp contrast to cells of pancreatic carcinoma lines studied, which were rapidly killed by the infection. Comparison of the intraperitoneal (IP) vs. the intravenous (IV) route of ADSC/MYXV administration revealed more pancreas-targeted distribution of the virus when ADSCs were delivered IP to mice bearing orthotopically injected PDAC. The biodistribution, tumor burden reduction and anti-tumor adaptive immune response were examined. Bioluminescence data, used to assess the presence of the luciferase-tagged virus after IP injection, indicated enhanced trafficking into the pancreata of mice bearing orthotopically-induced PDAC, as compared to tumor-free animals, resulting in extended survival of the treated PDAC-seeded animals and in the boosted expression of key adaptive immune response markers. We conclude that ADSCs pre-loaded with transgene-armed MYXV and administered IP allow for the effective ferrying of the oncolytic virus to sites of PDAC and mediate improved tumor regression.

## 1. Introduction

The incidence of pancreatic ductal adenocarcinoma (PDAC) has risen in the last 50 years and it is forecasted to be the second leading cause of cancer deaths within a decade. The dismal prognosis results from lack of specific early symptoms and early diagnostic methods. Patients usually present in the advanced stage of the disease and experience the failure of traditional anticancer therapies. Long-term disease-free survival is possible but the majority of diagnoses are unresectable cases, with an expected survival of less than 6 months. Modest curative benefits are offered only by early surgery with subsequent adjuvant therapy. Systemic recurrence after treatment suggests early metastasis in the course of pancreatic tumorigenesis, long before disease diagnosis [1].

The therapeutic failure in PDAC can be attributed to specific tumor cell biology, as well as features of the tumor milieu, both contributing to the high malignancy of this cancer. The tumor microenvironment of PDAC indeed presents a formidable challenge to therapy. Considerable desmoplastic stroma can constitute up to 90% of the tumor volume and are believed to originate from cancer-associated fibroblasts (CAFs) [2]. These are accountable for the chemoresistance of pancreatic tumors, by creating physical barriers that shield tumor cells from systemically injected therapeutic compounds. The heavy extracellular matrix within pancreatic cancer distorts tissue architecture and causes an abnormal structure of blood and lymphatic vasculature. The ensuing hypoxia is another hallmark of the PDAC microenvironment, associated with desmoplasia. It originates from desmoplasia-associated hypovascularization and, vice versa, favors desmoplastic progression by activating pancreatic stellate cells [3,4,5]. Desmoplasia and hypoxia are also barriers to the infiltration of both regulatory and effector lymphocytes, as well as to T cell activation [6,7,8]. Macrophages that are recruited adopt an immunosuppressive, pro-angiogenic M2-like state, block CD4+ T cell entry into the PDAC microenvironment, support PDAC progression, and thus are a marker of negative clinical prognosis [5,9,10].

The repertoire of treatment strategies in PDAC has been expanding in the last decades to include novel approaches such as targeted drugs, non-coding RNAs (miRNAs) and immunotherapy [11]. However, targeted chemotherapeutics have only moderately improved PDAC outcomes and have not altered 5-year survival. Gemcitabine and erlotinib, used to treat advanced disease, both yield only a modest clinical benefit. The development of chemoresistance and the pro-metastatic propensity of pancreatic cancer are related to the transition from epithelial to mesenchymal cells (EMT) [12,13]. Gene expression data have demonstrated the contribution of EMT cells to chemoresistance in PDAC [13,14].

Overall, the prevailing notion is that dense stroma, multiple genetic mutations, compensatory alternative pathways and aggressive metastatic spread cause PDAC chemoresistance, even to multidrug regimens. These factors are also at the root of the moderate success in targeting cancer-associated molecular pathways and the adverse effects linked to activated downstream effectors [11].

The ineffective nature of existing treatments for PDAC has stimulated the search for innovative therapeutic strategies. Oncolytic virotherapy is becoming increasingly sought-after for the treatment of many different neoplasms, including pancreatic cancer. The therapeutic effectiveness of oncolytic viruses (OVs) is the result of direct oncolysis, virus spread to adjacent cancer cells, and the elicited anti-tumor immune response.

Oncolytic viruses (OVs) are replication-competent and selectively target tumor cells, but they are not able to bind and/or productively replicate in most normal somatic cells. OVs can be genetically engineered to express inserted foreign transgenes to induce cancer cell death in a fashion that can stimulate anti-tumor immune responses [15]. OV replication in cancer cells yields infectious progeny that may further spread and reduce tumor burden [16].

Myxoma virus (MYXV) is a poxvirus with attractive safety profile for oncolytic therapy. MYXV infects and produces symptoms only in rabbits. Although MYXV is nonpathogenic to humans and mice, the virus exhibits natural tropism for a wide spectrum of human cancers [17]. MYXV has a 160-kb double-stranded DNA genome that has the capacity to accept numerous transgenes and is easy to manipulate. In this study, we armed oncolytic MYXV with the murine Light gene, encoding mouse tumor necrosis factor ligand superfamily member 14 (TNFSF14, synonym: LIGHT), which was designed to promote the influx of T lymphocytes into the tumor and thus intensify the immune response directed against the tumor. This ought to help stimulate the transformation of the “cold” tumor microenvironment, with a small number of effector immune cells, into a “hot” environment, with increased infiltration of other immune cells and cytokines. LIGHT is an inducible lymphotoxin which competes with HSV glycoprotein D for binding to a herpesvirus entry mediator (HVEM) expressed on T lymphocytes. LIGHT is expressed on activated T cells, natural killer (NK) cells, immature dendritic cells (DCs) and on the tumor and in the stroma [18]. LIGHT is an immune stimulator that contributes to the anti-tumor immune response, and its expression in the TME is associated with improved overall survival and relapse-free survival [19]. LIGHT–HVEM interaction has been shown to induce apoptosis of several tumor cell lines directly [20]. Therefore, LIGHT is a promising biotherapeutic adjuvant agent for cancer immunotherapy with OVs [21].

Systemic delivery represents the desired route to reach poorly accessible sites or disseminated metastatic lesions. IV administration of naked OVs is, however, a very inefficient strategy for delivery. Rapid elimination from the bloodstream by antiviral defense mechanisms is the major obstacle to achieving the effective transfer of the viral cargo to distant tumor sites. IV delivery of a viral construct to tumor sites can be improved by exploiting protective carrier cells, e.g., [22]. Adipose-derived stem cells (ADSCs) of mesenchymal origin provide a unique cell carrier platform, where the virus can be at least partly protected from immune clearance pathways before delivery to the tumor site. In addition, ADSCs show natural chemotactic tropism for cancer tissues. However, in cases where IV delivery of cell-protected OV would not be effective (due to the “first pass” effect in the lungs) [23], the transfer of oncovirus cargo to a defined and identifiable tumor site can be accomplished through locoregional injection. In this study, we investigated whether carrier cells could also be beneficial for the delivery of OVs to PDAC via the intraperitoneal (IP) route. IP administration remains an interesting alternative when targeting OVs to abdominal-area cancer targets. Comparison of the two modes of OV delivery using a peritoneal murine tumor model revealed [24] that IP was associated with narrower biodistribution (a reduced frequency of virus detected in the kidney, lung and heart), decreased toxicity and greater therapeutic efficacy against peritoneal metastases. Tumor burden was more effectively reduced with IP, compared with IV administration. Median survival following IP administration was approximately twice that observed with IV administration.

Wennier et al. tested the use of unarmed recombinant vMyx-tdTr to treat disseminated pancreatic adenocarcinoma in the peritoneal cavity of immunocompetent mice. They showed that oncolytic therapy, followed by gemcitabine, could decisively improve animal survival [25]. In our study, we assessed the usefulness of the novel armed MYXV construct (vMyx-mLIGHT-Fluc/tdTr) in experimental therapy on orthotopically-induced murine PDAC via IP delivery of the viral cargo using the mesenchymal ADSC platform.

## 2. Materials and Methods

### 2.1. Recombinant Viruses

Recombinant MYXV constructs (vMyx-EGFP, vMyx-EGFP/tdTr and vMyx-mLIGHT/FLuc/tdTr) derived from the wild-type Lausanne strain of myxoma virus (vMyx-WT) were used. The recombination cassettes were inserted in the intergenic region between the M135 and M136 open reading frames (ORFs). vMyx-EGFP expresses EGFP from the early/late promoter; the vMyx-EGFP/tdTr tandem system allows the expression of EGFP at both early and late infection stages (early/late promoter), whereas tdTr is expressed at the late infection stage (poxvirus p11 late promoter) [26]. The vMyx-mLIGHT-FLuc/tdTr construct expresses mouse LIGHT and firefly luciferase at both the early and late infection stages (early/late promoter) and tdTr under a synthetic poxvirus late promoter. vMyx-mLIGHT-FLuc/tdTr was constructed by inserting a DNA cassette containing the coding sequences of murine LIGHT (TNFSF14, see Supplementary Materials for the nucleotide sequence and Figure S1), firefly luciferase and tdTr at an intergenic location between the M135 and M136 genes in the wild-type MYXV strain Lausanne genome. A recombinant plasmid was constructed using the Gateway System (Thermo Fisher Scientific, Waltham, MA, USA) [17]. The sequence encoding murine LIGHT was PCR-amplified using gene-specific primers with the forward primer containing the synthetic early/late (sE/L) promoter sequence. The resultant sE/L-LIGHT fragment was PCR-ligated to another PCR fragment containing the MYXV M135 gene. The resultant attB1-M135-sE/L-LIGHT-attB4 PCR fragment was recombined into the pDONR221-P1P4 plasmid (Invitrogen, Waltham, MA, USA) using the BP clonase enzyme mix (Invitrogen). The construction of the plasmid having tdTr (attB4r-p11tdTr-attB3r) and another plasmid having FLuc and the MYXV M136 gene (attB3-sE/L-FLuc-M136-attB2) was described previously [27]. All three plasmids, along with the pDEST40 destination plasmid (Invitrogen) were then subjected to an LR recombination reaction using LR clonase II (Invitrogen) to generate the final plasmid construct. The recombinant virus was then created using the method described previously for making recombinant MYXV [17].

### 2.2. Virus Purification and Titration

MYXVs were produced in RK13 cells (at multiplicity of infection /MOI/ = 0.1). When the cytopathic effect was visible (ca. 72 h; ±80% confluency), cells were harvested, centrifuged (1500 rpm, 10 min, 4 °C), resuspended in 10 mM Tris–HCl (pH = 8), and subjected to three freeze/thaw cycles and cup sonication (5 × 1 min). Cell debris was removed by centrifugation. Homogenates containing the virus were layered onto the 36% sucrose cushion and ultracentrifuged (10^5^ × g/1 h, 4 °C). The supernatant was removed, and the pellet resuspended in 10 mM Tris–HCl (pH = 8). The quantity of infectious viral particles was determined by titration on RK13 cells (4 × 10^5^/well; 6-well plate). After 3–4 days, fluorescent foci were counted using an inverted microscope (Leica Microsystems, Mannheim Germany). Viral titer (focus-forming units per milliliter (FFU)/mL) was calculated as the number of foci multiplied by the dilution.

### 2.3. Cell Lines

Human (Panc–1, AsPC–1) and murine (Pan02) pancreatic ductal adenocarcinoma cell lines were used. The human lines were from ATCC; the murine line was a gift from GMF. Rabbit RK13 kidney epithelial cell line (ATCC) was used to propagate MYXV constructs. Cells were maintained in DMEM (RK13, Panc-1 and Pan02) or RPMI-1640 (AsPC–1) media, both supplemented with 10% FBS (EURx) and 1% penicillin-streptomycin (Sigma-Aldrich, Poznan, Poland) Cultures were grown in a humidified 5% CO_2_ incubator at 37 °C and were routinely tested for mycoplasma contamination.

### 2.4. ADSCs Isolation and Culture

Fragments of adipose tissue obtained on-site (MSC National Research Institute of Oncology) from human donors (Table S1) were washed with PBS^−^ (Gibco™, ThermoFisher Scientific, Warsaw, Poland) containing 1% FBS (EURx) to remove contaminating hematopoietic cells. The tissue was cut into small pieces and digested using collagenase type I solution (200 U/mL, Gibco) and shaking (1 h/37 °C/180 rpm). Cell suspensions were centrifuged (1500 rpm/10 min), filtered through 70-μm and 40-μm strainers and seeded in culture flasks using MEM (Sigma-Aldrich, Poznan, Poland) supplemented with 10% human platelet lysate (Sigma), heparin (2 U/mL, Polfa), 1% non-essential amino acids (Gibco) and 1% penicillin–streptomycin (Sigma). After 72 h, cultures were washed with PBS^−^ to remove non-adherent cells and rinsed with fresh culture medium. Sub-confluent cultures were split at a 1:3 ratio. Differentiation into adipocytes, osteocytes and chondrocytes was analyzed (passage 3) using the Human Mesenchymal Stem Cell Functional Identification Kit (SC006, R&D Systems), containing Goat Anti-Mouse FABP-4 Antigen Affinity-purified Polyclonal Antibody (adipocyte marker), a Mouse Anti-Human Osteocalcin Monoclonal Antibody (osteocyte marker) and Goat Anti-Human Aggrecan Antigen Affinity-purified Polyclonal Antibody (chondrocyte marker). Cells were stained with Biotinylated Rabbit Anti-Goat IgG (immunoglobulin G) Antibody (H + L), Texas Red Streptavidin (Vector Laboratories, BA-5000 and SA-5006, respectively) or Goat Anti-Mouse Alexa Fluor Plus 488 secondary antibody (Thermo Fisher Scientific, No. A32723). Nuclei were counterstained with DAPI (Thermo Fisher Scientific, No. 62248). The morphology of adipose-derived stem cells was inspected using the Zeiss LSM 710 confocal microscope system.

### 2.5. Flow Cytometry Analysis of ADSC Phenotype

To confirm the phenotype of ADSCs, cultured cells were incubated with appropriate antibodies (20 min/RT), rinsed with and resuspended in Cell Wash Buffer (BD Biosciences). Analysis was performed using a BD FACS Canto II flow cytometer (Becton-Dickinson). The phenotype of both uninfected and infected ADSCs (vMyx-WT; MOI = 5) was analyzed. The positive cell population gates were set using isotype IgG controls. The presence of ADSC-associated surface markers (CD73, CD90, and CD105) and the co-occurring absence of blood cell-lineage-specific markers (CD11b, CD19, CD34, CD45 and HLA-DR) was examined using the Human MSC Analysis Kit (BD Biosciences, No. 562245).

### 2.6. Infectiveness of RK13, ADSC and Pancreatic Cell Lines to MYXV

ADSCs, RK13 and three pancreatic adenocarcinoma cell lines (murine Pan02, human Panc-1 and AsPC-1) were tested for susceptibility to MYXV infection. Cultured cells (2 × 10^5^ cells/well; 6-well plate) infected with vMyx-EGFP (MOI = 5) were collected (24, 48 and 72 h p.i.), centrifuged (2000 rpm/2 min), washed twice and resuspended in PBS^−^ (200 µL). Cell aliquots were incubated with 7-aminoactinomycin D (7-AAD; 5 µL) for 2 min to determine viability and analyzed for enhanced GFP expression using flow cytometry (BD FACS Canto II). 7-AAD emission was detected using a long-pass filter (670 nm), and a region for live cells was defined. Non-infected cells were used as a control.

### 2.7. Expression of Early and Late MYXV Genes

To visualize early and late gene expression, ADSCs, RK13 and three pancreatic adenocarcinoma cell lines (murine Pan02, human Panc-1 and AsPC-1) were infected with vMyx-EGFP/tdTr (MOI = 5). Cells were plated into 4-well chamber slides (5 × 10^4^ cells/well). After 24 h p.i., cells were washed with PBS^−^ and fixed in paraformaldehyde (4%) for 10 min at room temperature (RT). Cell nuclei were stained with DAPI Counterstain (Life Technologies™, ThermoFisher Scientific, Warsaw, Poland). Infection was evaluated using fluorescence microscopy (Zeiss LSM 710 confocal workstation).

### 2.8. Single-Step Growth Analysis of Viral Replication in Cell Cultures

Cultures (5 × 10^4^ cells/well; 24-well plate) of ADSCs, RK13 and three pancreatic cancer cells lines (murine Pan02, human Panc-1 and AsPC-1) were infected (in triplicate) with vMyx-mLIGHT/Fluc/tdTr (MOI = 5). The inoculum was removed at 90 min post-infection and cells were further incubated (5% CO_2_, 37 °C) with fresh medium. Next, cells were trypsinized and collected (at 3-, 6-, 12- and 24-h time points). Following centrifugation (2000 rpm/2 min), cells were resuspended in 200 µL hypotonic swelling buffer (5 mL of 1M Tris-HCl (pH = 8.0) and 1 mL of 1M MgCl_2_) and frozen at −80 °C. Before titration, cells were thawed and sonicated (2 × 1 min) to disaggregate virus complexes. Samples from the examined time points were titrated back onto RK13 cells (4 × 10^5^ cells/well; 6-well plate) by serial dilution. Foci were counted using an inverted fluorescent microscope (Leica Microsystems). Titers (FFU/mL) were calculated (number of foci multiplied by the dilution factor).

### 2.9. Cytotoxicity of MYXV for ADSCs, RK13 and Pancreatic Cancer Cell Lines

To assess cytotoxic effects of MYXV infection on ADSCs, RK13 and three pancreatic cancer cell lines (murine Pan02, human Panc-1 and AsPC-1) cell cultures (1 × 10^4^ cells/well; 96-well plate) were infected with vMyx-mLIGHT/Fluc/tdTr at increasing MOIs (0.1, 1, 5 and 10). After 24 and 48 h, cell viability was evaluated using an MTS assay (CellTiter 96^®^ AQueous Non-Radioactive Cell Proliferation Assay kit; Promega) and a Biotek plate reader (490 nm).

### 2.10. Animal Care

All animal procedures were performed in accordance with European Union (EU) law, after approval by the Local Ethics Committee, Medical University of Silesia, Katowice, Poland. Six-to-eight-week-old C57Bl/6NCrl female mice (total *n* = 235; Charles River Laboratories) were used. Animals (18–22 g) were housed in HEPA-filtered IVC System cages (Allentown Caging Equipment) under a controlled dark/light cycle (12 h/12 h) and were fed a pathogen-free standard diet (Altromin 1314) and water ad libitum. All efforts were made to minimize animal suffering.

### 2.11. Orthotopic Tumor Implantation

For orthotopic tumor implantation, C57Bl/6NCrl female mice (subtotal *n* = 163) were anesthetized with isoflurane (1–3% vol.) and injected with carprofen (5 mg/kg, ScanVet) into the nape of the neck. The surgical field was sterilized with iodine and a ca. 1-cm-long incision was made beside the splenic silhouette. With the entire pancreas and spleen exposed, Pan02 cancer cell suspension was slowly injected into the pancreatic head area using a 27G needle, following which the pancreas and spleen were pushed slightly back into the abdominal cavity and the abdominal muscle layer was closed with a single continuous 4-0 polysorb suture. The skin incision was finally closed with an autoclip wound closing system and buprenorphine (0.03 mg/mL) was administered to assist recovery. Animal health was monitored daily. Only single animals from control groups reached termination criteria. Euthanasia was conducted by means of cervical dislocation.

### 2.12. Orthotopic Injection of Pan02-luc Cells with Simultaneous Administration of ADSCs Pre-Infected with MYXV

C57Bl/6NCrl mice (*n* = 6/group) were orthotopically injected with the Pan02 cells (1 × 10^6^ cells/25 µL PBS^−^), followed by immediate administration of ADSCs (5 × 10^5^ cells/25 µL PBS^−^) pre-infected (MOI = 5) with vMyx-mLIGHT/Fluc/tdTr. As controls, non-infected ADSCs, unshielded vMyx-mLIGHT/Fluc/tdTr (5 × 10^5^ FFU/25 µL PBS^−^) or PBS^−^ alone were used. After 21 days, the mice were sacrificed, and pancreata and spleens were excised, weighed and measured for size.

### 2.13. Bioluminescence Imaging (BLI) of MYXV Distribution Following Administration to Mice

C57Bl/6NCrl mice (*n* = 3/group) were orthotopically implanted (day 0) with Pan02 cells (1 × 10^6^/30 µL PBS^−^) (designated the +Pan02 group), or with 30 µL PBS^−^ for unchallenged mice (referred to as the −Pan02 group). Seven days after implantation, the mice were injected intraperitoneally (IP) with either a single dose of ADSCs previously infected (MOI = 5) for 24 h with vMyx-mLIGHT-Fluc/tdTr (5 × 10^5^ cells/100 µL PBS^−^), or with unshielded vMyx-mLIGHT-Fluc/tdTr (5 × 10^5^ FFU/100 µL PBS^−^). Bioluminescence imaging (BLI) was performed using the Lumina IVIS Imaging System (PerkinElmer). At various time points (3–96 h) after delivery of luciferase gene-carrying MYXV, the mice were injected IP with 1.5 mg D-luciferin (Promega). BLI data were acquired and regions of interest (ROIs) determined in both intact animals and dissected organs (pancreas, spleen, liver, lungs, heart and leg muscle).

### 2.14. Therapy of Immunocompetent Mice Bearing Orthotopic Pancreatic Tumors

Pancreatic tumors were established in recipient C57Bl/6NCrl mice (*n* = 10–11/group) (day 0) by orthotopic implantation of 1 × 10^6^ Pan02 cells/30 µL PBS^−^. For five-dose treatment regimens (days 4, 8, 12, 16 and 20), mice were injected IP with ADSCs infected (MOI = 5) for 24 h with vMyx-mLIGHT-Fluc/tdTr (5 × 10^5^ cells/100 µL PBS^−^) or with unshielded vMyx-mLIGHT-Fluc/tdTr (5 × 10^5^ FFU/100 µL PBS^−^) or 100 µL PBS^−^ (control). After 21 days, mice (*n* = 3/group) were sacrificed, peripheral blood was collected, and the pancreas, spleen and liver were excised, weighed and measured for size. Peripheral blood and pancreatic tissue were used for flow cytometry studies, whereas pancreas, spleen and liver tissues were formalin-fixed and used for histological assessments. The remaining treated mice (*n* = 7–8/group) were monitored for survival.

### 2.15. Histological Assessments

Formalin-fixed and paraffin-embedded pancreas, spleen and liver sections (5 µm-thick) were H&E stained, scanned and analyzed microscopically using a digital slide scanner and CaseViewer software (3D HISTECH, Budapest, Hungary) by an experienced pathologist. Masson’s staining was performed to assess the amount and localization of connective tissue in the tumor structure. Mitotic Index [28] was rated under 400× magnification (40 × 10) for 10 high power fields (HPF; field number 26.5).

### 2.16. Flow-Cytometry Analysis of CD4, CD8, CD3 Tumor-Infiltrating Lymphocytes

Pancreatic tumors were established orthotopically, and five-dose treatment conducted as described earlier. After 21 days, mice (*n* = 3) were sacrificed, peripheral blood samples were collected in EDTA-coated tubes, and pancreata were excised. Blood samples were treated with Red Blood Cell Lysis Buffer (BD Biosciences). Single-cell suspensions derived from pancreas tissue were obtained using a digestion mix containing 250 U/mL collagenase Type I (Gibco), 0.2 mg/mL hyaluronidase type IV-S (Sigma-Aldrich) and 0.02 mg/mL DNase I (Worthington) in DMEM + 10% fetal bovine serum (EURx). The digested samples were mashed through a sterile 70-µm nylon mesh cell strainer into ice-cold PBS^−^ containing 1% FBS. Red blood cells in pancreatic digests were lysed (3 min) on ice using ACK Lysis Buffer (Lonza) and passed through a 40-µm nylon mesh cell strainer. To quantify percentages of CD4+, and CD8+ populations in pancreas and blood, the generated samples were treated with antibodies (20 min/RT). After fluorescent labeling, 4 × 10^4^ cells were washed and analyzed using flow cytometry (BD FACS Canto II). The following monoclonal antibodies were used according to the manufacturer’s instructions: PerCP/Cyanine5.5 anti-mouse CD45 (clone 30-F11; BioLegend, San Diego, CA, USA), phycoerythrin (PE) anti-mouse CD3 (clone 17A2; BioLegend), fluorescein isothiocyanate (FITC) anti-mouse CD4 (clone GK1.5; BioLegend) and APC/Cyanine7 anti-mouse CD8a (clone 53-6.7; BioLegend).

### 2.17. Statistical Analysis

Graphs were plotted using GraphPad Prism 7 (GraphPad Software, San Diego, CA, USA) Statistical differences were determined using a one-way ANOVA test, followed by Tukey’s multiple comparisons test or two-way ANOVA with Tukey’s multiple comparisons test. Bartlett’s test was performed to ensure the suitability of the data for parametric significance tests. Kaplan–Meier survival curves were compared statistically using a log-rank test (Mantel–Cox). Data are presented as bars indicating means (±SD). The significance levels are indicated with asterisks: * *p* ≤ 0.05; ** *p* ≤ 0.01; *** *p* ≤ 0.001; **** *p* ≤ 0.0001. *p*-values < 0.05 were considered statistically significant.

## 3. Results 

### 3.1. Identity of Human Mesenchymal Adipose Tissue-Derived Stem Cells (ADSCs)

**Figure 1:**
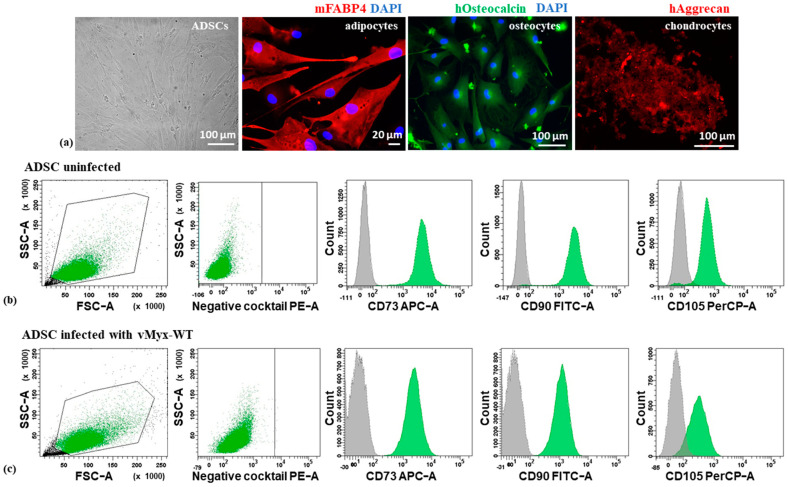
Characterization of adipose tissue-derived stem cell ADSC (**a**) morphology (far left) and differentiation of ADSCs into adipocytes (anti-mFABP4 antibody), osteocytes (anti-hOsteocalcin antibody) and chondrocytes (anti-hAggrecan antibody, red); DAPI-counterstained nuclei (magn. 40×; scale bar = 20 µm for adipocytes; magn. 20×; scale bar = 100 µm for osteocytes and chondrocytes). Flow cytometry plots of uninfected ADSCs (**b**) or ADSCs infected (**c**) with vMyx-WT (MOI = 10) confirming the presence of CD73, CD90 and CD105 and the concomitant absence of CD11b, CD19, CD34, CD45 or HLA-DR markers; gray: isotype control; green: viable ADSCs. Gating parameters based on the signal of isotype IgG control probes.

The isolated primary human cultured ADSCs were characterized (Figure 1a–c) by morphology, multipotency and the combination of positive as well as negative surface markers. ADSCs showed fibroblast-like morphology and their ability to differentiate into mature cells of other tissue types, specifically adipocytes, osteocytes and chondrocytes, was confirmed (Figure 1a). Using an MSC Functional Identification Kit and flow cytometry, the immunophenotype of virus-uninfected ADSCs (Figure 1b) or unarmed wild-type MYXV (vMyx-WT)-infected ADSCs (Figure 1c) was investigated and confirmed the presence of CD73, CD90 and CD105 (human ADSC-associated surface markers), and the concomitant absence of CD11b, CD19, CD34 and CD45 (blood cell lineage-specific markers), as well as the lack of HLA-DR (MHC Class II receptor). We also confirmed the expression of MYXV-encoded LIGHT in ADSCs infected with vMyx-mLIGHT-Fluc/tdTr construct and the absence of its expression in ADSCs infected with vMyx-WT and non-infected (Figure S2). The Light gene transcript was measured using RT-qPCR and expression was rendered as a ratio of the target gene (Light) vs. the reference gene (glyceraldehyde 3-phosphate dehydrogenase/GAPDH/). The distribution of the LIGHT protein in cells infected with vMyx-mLIGHT-Fluc/tdTr was also confirmed in the cytoplasm and cell surface (Figure S3).

### 3.2. In Vitro Infection and Replication of MYXV in Pancreatic Ductal Adenocarcinoma Is Cell-Type-Dependent

**Figure 2:**
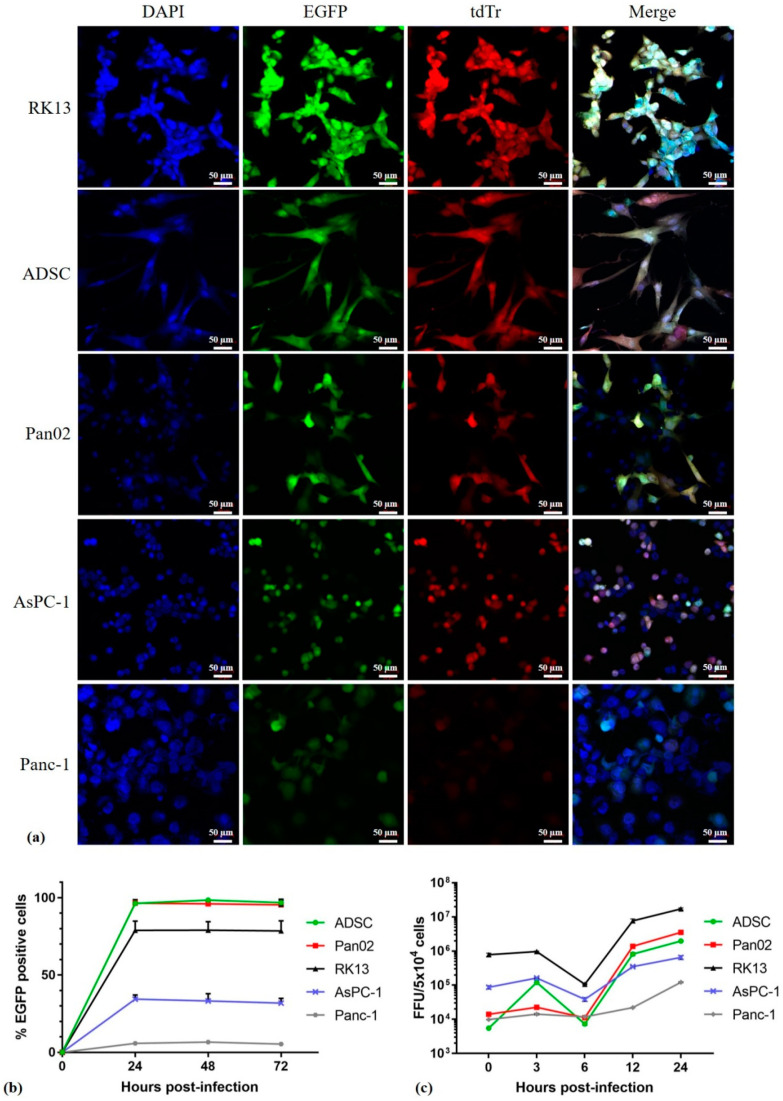
MYXV infection and replication in pancreatic cancer cells, RK13 and ADSC. Cultures of ADSCs, Pan02, RK13, AsPC-1 and Panc-1 cells were infected: (**a**) with vMyx-EGFP/tdTr (MOI = 5). At 24 h post infection (p.i.) the infection was visualized by fluorescence microscopy (magn. 20×; scale bar = 50 µm; Zeiss LSM 710 confocal Workstation); blue: DAPI staining (nuclei); green: EGFP fluorescence; red: tdTr fluorescence; (**b**) with vMyx-EGFP (MOI = 5), collected at the indicated time points and analyzed by means of flow cytometry to determine the percentage of infected EGFP-positive cells; (**c**) with vMyx-mLIGHT-Fluc/tdTr (MOI = 5) to generate single-step growth curves. Cells were collected at the indicated time points and lysed to determine viral titers. Titers for each sample were performed in triplicate; error bars shown are means ± SD.

MYXV is a potential OV candidate for the treatment of many human cancers. A panel of cell lines was tested for permissiveness to infection with unarmed vMyx-EGFP/tdTr, vMyx-EGFP (Figure 2a,b). Three pancreatic ductal adenocarcinoma cell lines (murine Pan02, human Panc-1, human AsPC-1), as well as primary human mesenchymal ADSC carrier cells and RK13 cells of rabbit origin used to propagate the virus were tested (positive control). Panc-1 and AsPC-1 harbor the mutant K-Ras gene, which is involved in the EGFR pathway. Pan02 is a non-metastatic murine line that is highly sensitive to gemcitabine, a drug widely used in pancreatic cancer treatment.

In order to better understand the permissiveness to MYXV infection in these cells, we compared the ability of the vMyx-EGFP/tdTr reporter construct expressing EGFP (enhanced green fluorescent protein; early/late expression) and tdTr (tandem dimer tomato red fluorescent protein; late expression only) or the vMyx-EGFP construct expressing only EGFP to infect and spread in different cell types from the tested panel. Cells were infected with the recombinant virus (MOI = 5) and evaluated by means of fluorescence microscopy (Figure 2a) or flow cytometry (Figure 2b). We observed significant differences in infection progression by the virus between the tested cell types. In RK13, ADSC and Pan02 cell lines, the MYXV construct appeared to initiate a permissive infection and underwent normal cell-to-cell spread. Fluorescence microscopy analysis revealed the expression of both EGFP and tdTr from vMyx-EGFP/tdTr and flow cytometry showed an increase in the percentage of infected EGFP-positive cells (Figure 2b). There was less MYXV infection observed in AsPc-1 and Panc-1 cell lines, which can be considered either semi-permissive or non-permissive for MYXV replication, although they did permit at least detectable levels of viral gene expression from early promoters. Flow cytometry showed a decreased percentage of infected EGFP-positive cells after 72 h p.i. (30% and 6%, respectively).

However, single-step growth curves showed that the recombinant vMyx-mLIGHT-Fluc/tdTr construct (LIGHT and Fluc—early/late expression; tdT—late expression only) used for the infection of AsPC-1 and Panc-1 cells did not produce new infectious virions, whereas the infection of RK13, ADSC and Pan02 cells with the same recombinant produced significantly more progeny virions (Figure 2c). The most notable differences were observed in Panc-1 cells, which did not support productive MYXV replication, as reported previously [29], despite allowing early gene expression in the initially infected cells (Figure 2c). Of note, human ADSCs and murine Pan02 cells showed the highest viral titers, an approximately 3.6-log and 3.2-log increase, respectively, at 24 h p.i. when compared to other tested cell lines. In rabbit RK-13 cells, the MYXV construct achieved a 1.7-log increase in titer. Of the panel of cell lines tested, human pancreatic cancer cell lines (AsPC-1 and Panc-1) were the least susceptible, with viral titers achieving approximately 0.17-log and 0.12-log increases, respectively, at 24 h p.i. Taken together, these results show that different types of pancreatic cancer cells were variably permissive for the MYXV construct tested, although all were capable of supporting at least detectable levels of early virus gene expression.

### 3.3. MYXV Infection Reduces the Viability of Pancreatic Cancer Cells In Vitro

**Figure 3:**
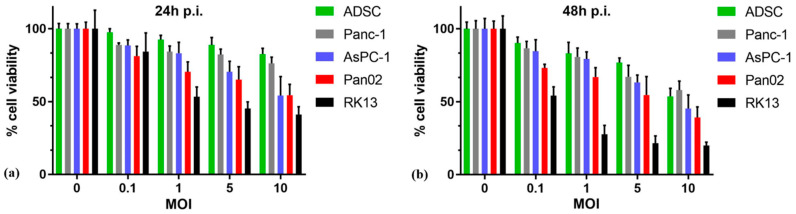
MYXV infection reduces the viability of pancreatic cancer cells. Pancreatic cancer cell (Panc-1, AsPC-1 and Pan02) ADSCs, as well as RK13 (1 × 10^4^ cells/well), were infected with vMyx-mLIGHT-FLuc/tdTr at four various MOIs (0.1; 1; 5 and 10) and analyzed for cell viability using the MTS assay at (**a**) 24 h and (**b**) 48 h post-infection. The assays were performed in triplicate; error bars shown are means ± SD.

The MTS cell viability assay was used (Figure 3a,b) to determine if infection with oncolytic MYXV construct vMyx-mLIGHT-FLuc/tdTr at different MOIs (0.1–10) would result in reduced viability in cultures of cells from the tested panel at 24 and 48 h p.i. Primary human ADSCs remained the most viable, irrespective of increasing values of MOI, as compared to other cell lines at both time points tested (almost 90% at 24 h and 77% at 48 h/MOI = 5). On the other hand, the highly MYXV-susceptible RK13 cells showed viability reduced to 41% at 24 h, and to 20% at 48 h at the MOI = 10. The murine Pan02 cancer cell line, as well as the human AsPC-1 cancer line, were only 54% viable already at 24 h at the MOI = 10, and 24 h later only 39% and 45% cells, respectively, remained viable. Human Panc-1 cells were the least susceptible to the cytotoxic effects of vMyx-mLIGHT-FLuc/tdTr infection and retained 82% viability at 24 h and 67% at 48 h/MOI = 5). Thus, the infection of cultured pancreatic adenocarcinoma cells with the vMyx-mLIGHT-FLuc/tdTr construct leads to a marked-to-significant reduction in cell viability, suggesting that oncolytic treatment of even the semi- and non-permissive types of cancers may provide sizeable therapeutic benefits in vivo, as well as still delivering therapeutic proteins such as LIGHT, if expressed under early virus promoter control. ADSCs seem to fulfill the criteria for an effective viral cargo carrier that is also capable of supporting viral replication and potentially delivering either parental or progeny virus into cancer sites.

### 3.4. Orthotopic Injection of Pan02 Cells with ADSCs Pre-Infected Ex Vivo with MYXV Inhibits Establishment and Growth of Pancreatic Cancer

**Figure 4:**
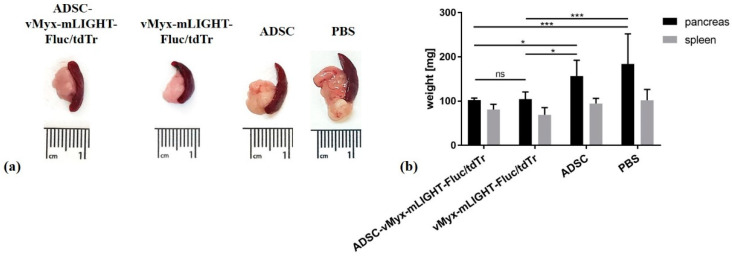
Inhibition of pancreatic adenocarcinoma formation after orthotopic implantation of Pan02 cells and consecutive injection of ADSC-shielded MYXV construct (vMyx-mLIGHT-Fluc/tdTr) or unshielded MYXV. (**a**) Macroscopic appearance of pancreas and spleen upon necropsy; (**b**) mass of pancreata and spleen (*n* = 6) after orthotopic injection (21 days). The data show means ± SD of two independent experiments. (* *p* ≤ 0.05; *** *p* ≤ 0.001; ns—not significant).

We examined if the oncolytic properties of MYXV could prevent the growth of pancreatic adenocarcinoma in mice following the orthotopic injection of murine cancer cells, under conditions in which the virus is co-delivered with the cancer cells (Figure 4). To do so, naïve immunocompetent mice were implanted orthotopically with Pan02 adenocarcinoma cells and then immediately treated by orthotopic injection of LIGHT-encoding MYXV construct (vMyx-mLIGHT-Fluc/tdTr), either unshielded or shielded by ADSC carrier cells (ADSCs that had been previously infected ex vivo with the vMyx-mLIGHT-Fluc/tdTr). As controls, ADSCs or PBS^−^ were used. Twenty-one days after this co-implantation procedure, the dissected pancreata were macroscopically inspected and both size and mass were recorded. The experiment revealed (Figure 4a) no tumor presence and no organ size increase if vMyx-mLIGHT-Fluc/tdTr constructs were injected (i.e., either ADSC-shielded or unshielded). Statistically significant differences were revealed (Figure 4b) between the masses of pancreata from control mice and pancreata from both virus-recipient mouse groups.

### 3.5. ADSC-Enhanced Biodistribution and Tumor Targeting of MYXV in Pancreatic Cancer-Bearing Mice after Intraperitoneal Injection

**Figure 5:**
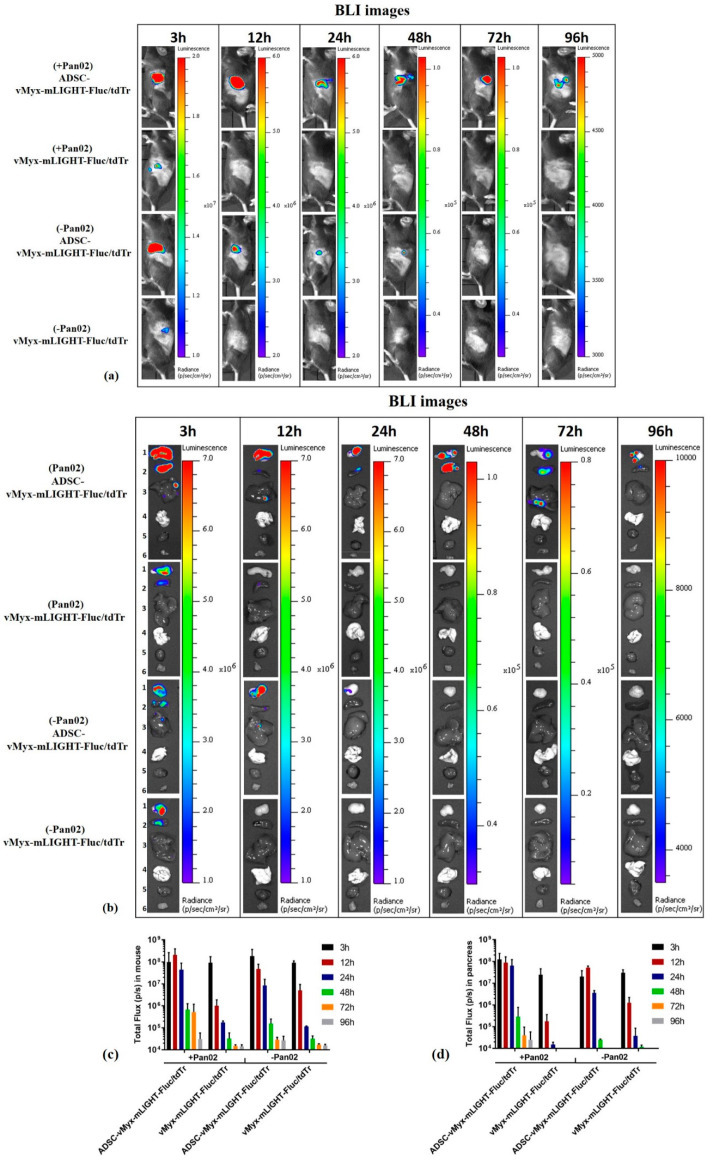
Effect of intraperitoneal injection of MYXV construct (vMyx-mLIGHT-Fluc/tdTr) on biodistribution in mice. Bioluminescence (BLI) images were acquired either in unchallenged (−Pan02) mice injected IP with PBS^−^ only, and in challenged (+Pan02) orthotopic pancreatic adenocarcinoma-bearing mice at various time points (3 h, 12 h, 24 h, 48 h, 72 h and 96 h) post-injection of ADSC-shielded or unshielded MYXV construct (vMyx-mLIGHT-Fluc/tdTr); (**a**) intact mice; (**b**) dissected organs (1-pancreas, 2-spleen, 3-liver, 4-lungs, 5-heart and 6-muscle). Region of interest (ROI)-based analysis of total photon flux in (**c**) intact animals and (**d**) dissected pancreata. BLI is expressed as radiance (photons/sec/cm^2^/sr). Different radiance scales are shown to cover the whole span of bioluminescence. The data show means ± SD of two independent experiments (*n* = 3).

Comparison of the intraperitoneal (IP) versus intravenous (IV) route of ADSC/MYXV administration revealed pancreas-targeted distribution of the virus when ADSCs were delivered IP to mice bearing orthotopically injected PDAC (Figure S4). Bioluminescence (BLI) data were acquired using IVIS (Figure 5) to assess the distribution of the ADSC-shielded or unshielded vMyx-mLIGHT-Fluc/tdTr construct, administered intraperitoneally after 7 days (single IP injection) into recipient mice either harboring tumor lesions (+Pan02) or free from tumor lesions (−Pan02). Intact animals (Figure 5a) and dissected organs (Figure 5b) were examined. Following injection, bioluminescence signals for both shielded and unshielded MYXV peaked within three hours in the pancreas, but were also present in the spleen and liver. The shielded virus was detected throughout the examined time span (96 h post-injection); later, it faded away. In contrast, no bioluminescence signal from the unshielded MYXV construct was detected in the pancreas after 48 h. No bioluminescence signal was observed in the lungs, heart or muscle tissue from any tested group.

The analysis of total photon flux data (Figure 5c) from intact mice bearing tumors (+Pan02) revealed that after 24 h p.i., the signal from the shielded virus was 200-fold stronger than that from its unshielded counterpart. However, after a further 24 h (48-h time point) the signal from the shielded virus decreased 100-fold, indicating either some transfer event following the release of the construct from ADSCs, or simply viral clearance. This signal, however, was still high at 72 h, contrary to that in unshielded MYXV which, at 72 h, was undetectable. The persistence of the shielded virus signal suggests a different fate of the actively expressing virus after its probable release from ADSCs, or possibly a source of signals other than the pancreas. In tumor-free mice (−Pan02) there was no essential difference in the kinetics of signal quenching between the shielded and unshielded virus, except that absolute signal values were generally somewhat lower for the unshielded construct. In these tumor-free mice, the signal from the shielded virus at 24 h was 100-fold higher than that of its unshielded counterpart, similar to tumor-burdened animals, again showing the protective nature of ADSCs. When signals from tumor-bearing and tumor-free mice were compared at the 24-h time point, a 4-fold difference was seen in favor of the shielded virus, suggesting increased targeting, perhaps due to the inflammatory nature of tumor foci, which attract ADSCs. The decrease of the shielded virus signal in tumor-free mice, occurring between 24 and 48 h, was similar to that seen in tumor-bearing mice and again suggests a gene expression-related event following the virus release in the tumor bed. However, the decrease was 50-fold, twice as small as that seen in tumor-bearing mice, and this difference could be ascribed to the presence/absence of tumor lesions. Analysis of total photon flux data for the dissected pancreata (Figure 5d) shows the kinetics of the signal decrease in tumor-bearing mice (+Pan02) to be generally similar to that in intact animals (Figure 5c), except that the signal for the shielded virus after 72 h was lower. This points to the presence of some other source of the signal (in addition to the pancreas) in intact animals, at least at this time point. The exceptionally large difference between shielded and unshielded virus signals from pancreata of tumor-bearing mice at 24-h time point (more than three orders of magnitude) demonstrates the effective shielding of the viral construct delivered by ADSCs to adenocarcinoma lesions. Finally, the comparison of signals from the shielded construct at the 48-h time point between pancreata dissected from tumor-bearing and tumor-free mice, shows a ca. 10-fold higher signal from the former; this further corroborates the virus-protective and tumor-targeting capacities of ADSC carrier cells.

### 3.6. LIGHT-Encoding MYXV Shielded by ADSC Enhances Anti-Tumor Immune Response and Improves Survival of Mice Bearing Orthotopic Adenocarcinoma Lesions

**Figure 6:**
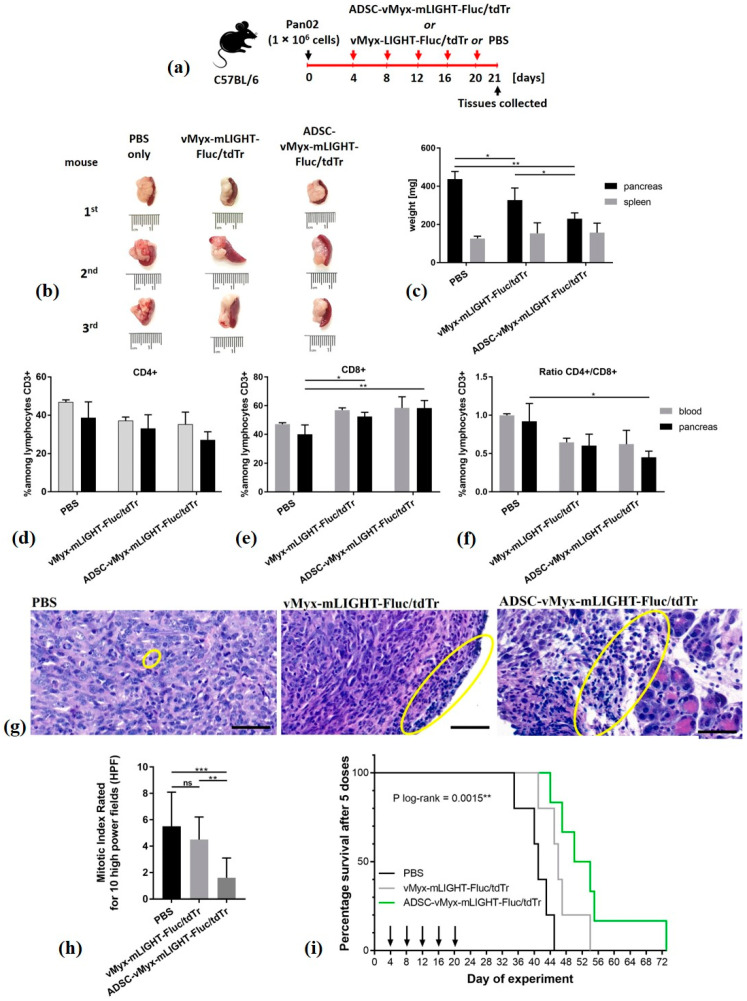
Therapy of experimental pancreatic adenocarcinoma using armed MYXV construct. C57Bl/6NCrl mice (*n* = 10–11) with induced orthotopic lesions were injected IP (days 4, 8, 12, 16 and 20) with LIGHT-expressing vMyx-mLIGHT-Fluc/tdTr, either ADSC-shielded or unshielded, or with PBS^−^; (**a**) timeline of experiment; (**b**) size of pancreata, 21st day; (**c**) weight of pancreata and spleen, 21st day; (**d**–**f**) flow cytometry data showing CD4+ and CD8+ cell percentages among CD3+ lymphocytes in the blood and pancreas; (**g**) histological appearance of representative H&E-stained sections (scale bars: 50 µm, magn. 48×): lymphocytic infiltrates: yellow ellipses, (**h**) mitotic index rated under 400× magnification for 10 high power fields (HPF); (**i**) mouse survival (*n* = 7–8): log-rank (Mantel–Cox) test; *p* = 0.0015. The data (mean ± SD) were analyzed with one-way ANOVA; statistically significant differences are indicated (* *p* ≤ 0.05; ** *p* ≤ 0.01; *** *p* ≤ 0.001; ns – not significant).

The endurance of human ADSCs infected with therapeutic MYXV following IP injection into immunocompetent mice was deemed sufficient to allow the survival of virus during bloodstream transit and its delivery to PDAC lesions before immune clearance. We thus examined the therapeutic effect of the LIGHT-expressing oncolytic MYXV construct (vMyx-mLIGHT-Fluc/tdTr) using immunocompetent mice (Figure 6). The animals were first engrafted orthotopically with murine Pan02 cells. Four days later, the mice received IP injections of vMyx-mLIGHT-Fluc/tdTr, either in unshielded or ADSC-shielded form. Control mice received PBS^−^ only. In total, five doses of the therapeutics were delivered (every 4 days), spanning three weeks of treatment (Figure 6a).

At the end of the therapeutic intervention (21st day) some animals were sacrificed and their pancreata and spleens inspected macroscopically for size and organ weight (Figure 6b,c). Pancreata from mice treated with ADSC-shielded MYXV revealed smaller sizes (Figure 6b), as well as reduced weight (Figure 6c) when compared to mice receiving unshielded MYXV or PBS^−^ (ca. 25% and 50%, respectively), suggesting a positive response to ADSC-mediated oncolytic therapy.

Samples of pancreatic tissue and blood were also examined by means of flow cytometry (Figure 6d–f) for signs of an adaptive anti-tumor immune response after the conclusion of therapy. The percentage of CD4+ helper cells (Figure 6d) among CD3+ lymphocytes for both pancreata and blood decreased following treatment with shielded and unshielded virus, but was not statistically significant. The percentage of CD8+ cells (Figure 6e) in murine blood increased following delivery of both shielded or unshielded virus, but not significantly either. Contrarily, the increase of CD8+ in pancreatic samples was significant for the shielded (*p* < 0.01) as well as the unshielded (*p* < 0.05) virus when compared to the PBS^−^ group. Changes in the CD4+/CD8+ ratio (Figure 6f) were only significant for pancreatic tissue samples and the shielded virus group (*p* < 0.05). Taken together, these changes suggest an enhanced immune response triggered by the oncolytic viral construct used and benefiting from using ADSCs as carrier cells.

H&E-stained tissue specimens (pancreas, spleen, liver) from both treatment groups and controls were microscopically evaluated by an experienced histopathologist. In pancreatic specimens from the group treated with unshielded MYXV, only minimal lymphocytic influx was usually present around the edge of tumor (Figure 6g, center panel). In contrast, pancreatic specimens treated with ADSC-shielded MYXV showed stronger lymphocytic infiltrates, especially in the peripancreatic adipose tissue (Figure 6g, right panel). Liver specimens revealed no pathology in the tissue architecture in any group.

The panels in Figure S5 (see Supplementary Materials) show additional micrographs of pancreatic and spleen sections from mice subjected to the therapeutic intervention with unshielded or shielded armed MYXV. Pancreata from animals treated with unshielded MYXV revealed spindle cell tumors, making up 25–60%; in the ADSC-shielded MYXV group the cellularity of tumor regions did not exceed 25% (panel 3). Both treated groups displayed large nuclear pleomorphism; oval, blunt-ended cell nuclei; no distinct nucleoli and fine-grained chromatin (panels 5–6). The unshielded MYXV group featured more atypical mitoses and spindle cells, with a hyperchromatic nucleus (panel 1). In the shielded MYXV group only single atypical mitoses and singly dispersed spindle cells were seen with a focal hyperchromatic nucleus (panel 6). The control group (PBS^−^) showed pancreas sections that featured spindle cell tumors invading acinar cells and surrounding interlobular ducts, large nuclear pleomorphism, oval blunt-ended cell nuclei, no distinct nucleoli, fine-grained chromatin and sinusoidal blood vessels (panels 1,4 and 10). In turn, micrographs of the spleen specimens from the group treated with unshielded MYXV showed spindle cell tumors covering the spleen (panel 14), whereas in the group treated with shielded MYXV only cancer cells with hyperchromatic nuclei between collagen fibers were visible, along with thickened capsules, inflammatory lymphocytes, as well as eosinophils (panel 15). Spleen specimens from the control group (PBS^−^) showed infiltration of spindle cell tumors adjacent to the spleen capsule (panel 13), whereas giant cells were scattered in the spleen parenchyma in all groups.

The mitotic index data (Figure 6h) for the pancreatic specimens show significantly decreased numbers of mitoses in the shielded MYXV group when compared to the PBS^−^ control group (*p* < 0.0001) and to the virus-treated group (*p* < 0.001).

The remaining mice from the therapeutic experiment were monitored and compared for survival with controls. The difference in survival (Figure 6i) between controls and mice that received five doses of unshielded MYXV was 42.8%, whereas that for ADSC-shielded MYXV was 88% (*p* = 0.0015). On the 54th day, 50% of animals which received ADSC-shielded MYXV were alive, compared to none in the unshielded construct group.

## 4. Discussion

PDAC presents a formidable challenge for oncological researchers, as the overall outcome of PDAC patients remains desperately poor. The characteristic feature of PDAC is its resistance against practically any treatment [30,31,32].

Oncolytic virotherapy represents a promising approach to augmenting the PDAC therapeutic repertoire. In the current manuscript, we report the results of an experimental therapy targeting orthotopically-induced PDAC lesions in immunocompetent mice using recombinant oncolytic MYXV encoding mouse tumor necrosis factor ligand superfamily member 14 (also called LIGHT), designed to boost the anti-tumor immune response induced by the virotherapy. The vMyx-mLIGHT-Fluc/tdTr construct was pre-adsorbed ex vivo onto adipose tissue-derived stem cells (ADSCs) of mesenchymal origin and subsequently infused into the peritoneal cavity of experimental animals that had been pre-seeded orthotopically with murine PDAC into the pancreas.

We show that the cultured human ADSCs had correct morphology and multipotency. In-vitro-expanded ADSC cells were shown to differentiate into adult cells of the expected downstream lineages. The phenotype of ADSCs, as assessed by characteristic surface markers and migratory properties, was also shown to remain unchanged between MYXV-infected ADSCs and uninfected ADSCs, at least in the 1–2 day interval post-infection. We confirmed the level of permissiveness of ADSCs and three tested pancreatic cancer cell lines to MYXV. We observed significant differences between these pancreatic cancer cell lines concerning the level of infection and replication of MYXV. In general, cancer cells can be either fully permissive (i.e., produce in excess of 10 infectious progeny virus units per cell), semi-permissive (i.e., produce the progeny virus at a level below 10 infectious units/cell) or non-permissive (no detectable progeny virus) to MYXV. All three categories of cells can nevertheless express MYXV-encoded transgenes controlled by early virus promoters, but only the first two types can also express transgenes under late viral promoter control. The in vitro results indicated that MYXV productively infects the murine pancreatic cancer cell line (Pan02) with evidence of a cytopathic effect, complete viral replication and increased cell death. However, levels of productive MYXV replication in human pancreatic cancer cell lines (AsPC-1 and Panc-1) showed that they were less susceptible to infection, and we define them as semi-permissive or nonpermissive for MYXV, respectively. ADSCs remained the most viable after MYXV infection, whereas RK13 (the control rabbit cell line used to propagate the virus) was highly MYXV-susceptible.

Despite the variable levels of progeny virus generated, infection of all cultured pancreatic adenocarcinoma cells with vMyx-mLIGHT-FLuc/tdTr led to a marked-to-significant reduction in cell viability. ADSCs, in contrast, support viral infection without rapid cell death, making this viral cell carrier useful for in vivo experiments where cell viability and taxis need only be maintained for a brief period in order to ferry the virus into cancerous sites, likely hours or less. The ability of cultured ADSCs to support MYXV replication is similar to that observed with bone marrow-derived mesenchymal stem cells [23]. This is a key feature of ADSCs, allowing the successful transfer of the virus into adjacent tumor cells via cell–cell contact. ADSCs thus seem to fulfill the criteria for an effective viral cargo carrier that is capable of transferring both the input (parental) and progeny virus to tumor cells, yielding sizeable therapeutic benefits in vivo.

We first showed that ADSCs infected with MYXV could effectively prevent the outgrowth of experimentally induced PDAC lesions when the cancer cells were co-implanted with MYXV or with ADSCs pre-infected ex vivo with MYXV. On the other hand, control tumors were rapidly induced in the absence of the virus. This pre-treatment experiment indicated that MYXV can be highly therapeutic against PDAC if the virus delivery to cancer cells in situ can be optimized. Thus, the model is a sensitive indicator of the efficiency of delivery of an OV to tumor cells at the pancreatic site of PDAC. Essentially similar outcomes seen with co-implantation of cancer cells and therapeutic constructs provide evidence of viral construct transfer from infected MSCs to cancer cells under in vivo conditions [23].

OV-mediated systemic therapy, for example after IV infusion of the unshielded virus, faces rapid and efficient anti-viral immune host responses, as well as other clearance obstacles. During bloodstream transit, the viral cargo is largely cleared in organs like the liver and spleen, although protective carrier cells can mitigate against this [22]. Whether IP administration of the tested therapeutic recombinant MYXV would be contingent upon, or benefit from, shielding by a protective cell carrier like ADSCs was an open question that we approached in this study. We report that IP administration of the protected viral therapeutic cargo by pre-loading onto ADSCs ex vivo yielded desirable pancreas-restricted distribution as compared to more standard IV administration. The significance of pre-loading carrier cells with oncolytic virus cannot be overestimated. Our data highlight the outcome of delivering such pre-loaded ADSCs. IVIS-generated images tracking the distribution of the viral construct (vMyx-mLIGHT-Fluc/tdTr) in PDAC-bearing mice following IP injection clearly demonstrate the delivery of the virus into the pancreas. The unshielded recombinant MYXV was rather rapidly cleared from the body after IP administration, as opposed to the virus shielded by ADSCs. The existing dogma postulates that anti-OV immune responses restrict viral replication and spread, and thus reduce direct OV-mediated killing of cancer cells. Therefore, the anti-tumor activity of the unshielded virus, although present, is likely not optimal therapeutically. This point is also illustrated by the difference in survival between the two therapeutic groups (MYXV only vs. ADSC-MYXV).

Photon flux IVIS data from intact mice demonstrate the advantage of shielding the virus using ADSCs and temporal signal changes suggest the release and transfer of the virus from carrier cells into target pancreatic cancer cells. The persistence of the signal from virus that had been pre-shielded with ADSCs implies either a different fate of the virus transferred from ADSCs or a source of the signal other than pancreas. Comparison of the signal between tumor-bearing and tumor-free control mice also confirms the protective benefits of ADSC pre-shielding and increased targeting in favor of tumor bearing mice, perhaps due to the inflammatory nature of tumor foci. Temporal differences in the signal are also suggestive of virus release and de novo infection of cancer cells, and signal differences between tumor-bearing and tumor-free animals could be ascribed to the presence or absence of tumor lesions. Total photon flux data, showing exceptionally large differences between shielded vs. unshielded virus signals from pancreata of tumor-bearing mice (several orders of magnitude), point to effective delivery and transfer of the viral construct to cancer cells within the pancreas. The potential ability of ADSCs to seek out pancreatic cancer cells and deliver oncolytic MYXV from an IP injection could be of great therapeutic value, especially when other delivery strategies are ineffective.

Ex-vivo-expanded hypoimmunogenic human adipose-derived stem cells (ADSCs) represent a unique delivery platform for OVs. They combine a natural capacity to home to inflammatory sites with a tumor tropism that is coupled with potent amplification of the OV load and transient suppression of anti-viral innate immunity, which hinders OVs from colonizing the tumor site and infecting cancer cells [33]. Importantly, IFNγ, which is involved in the induction of an anti-viral state, also modulates immunosuppressive features of ADSCs, counteracting anti-viral immunity. Thus, the dual capacity of ADSCs to offset the innate and adaptive arms of anti-viral immunity and to allow viral load amplification potential is conducive for the success of oncolytic virotherapy. In studies involving mice and repetitive treatment in which the access route is challenging, IP delivery remains an alternative to IV when the latter strategy is clearly ineffective, as it generally is for PDAC. Targeting the pancreas with an OV using mesenchymal stem cells as carriers is not nearly as effective via IV delivery, for the same reason that targeting lung neoplasias in this manner is ineffective—the “first pass” effect [23]. Although IP administration of pharmacological agents is minimally used in the clinic (mostly for the treatment of peritoneal cancers), in experimental animals it is a justifiable route for proof-of-concept studies where the goal is to evaluate the effect(s) of target engagement rather than the properties of a drug formulation and/or its pharmacokinetics for clinical translation [34].

Following the postulated release of the virus from ADSCs at the targeted tumor site, some intratumoral antiviral events occur, resulting in a “cold” to “hot” transition, reversal of immunosuppression and recruitment of immune cells. Cancer cell death-associated signals further contribute to the development of a tumor-specific adaptive immune response. Overall, antiviral innate and adaptive responses targeting virus replication sites target cancer cells since OVs preferentially infect cancer cells [35]. A decisive factor in successful immunotherapy is the presence of T cells and the reactivation of their anti-tumor properties. Even though T cells are rather low in PDAC, some data suggest that the tumor microenvironment (TME) mainly consists of various cellular components and the extracellular matrix and is highly immunosuppressive. Tumor-infiltrating lymphocytes are one of the crucial players in the TME of pancreatic cancer. On the other hand, although numbers of T cells in PDAC appear low [36], the tumor-reactive T-cell repertoire was found to be similar to that in melanoma, in which immunotherapy does show a therapeutic impact [37]. Since strong intra-tumoral CD8+ T cell infiltration is associated with prolonged survival, induction of anti-tumor T cell responses can indeed be a promising approach for PDAC [38,39,40].

In a previous study, we showed that the therapy targeting experimentally induced lung melanoma in mice with IL-15-encoding MYXV construct (vMyx-IL15Rα-tdTr) delivered by MSCs was effective and was able to reduce the tumor burden as well as triggering the inflow of CD8+ cells [33]. Here, we demonstrate another ADSC-shielded recombinant MYXV that expresses LIGHT protein, used to extend the survival of treated mice and to increase the influx of T lymphocytes into the tumor.

Following a five-dose therapy of orthotopic PDAC lesions with ADSC-vMyx-mLIGHT-Fluc/tdTr, we have been able to show a reduced tumor burden effect, suggesting a positive response to treatment. At the end of the therapeutic intervention (21st day), some animals were thoroughly inspected post-mortem for any signs of macroscopic pathologies and none were found, except for remaining tumor lesions. Scars surgically-induced at the onset of the experiment were healed.

An adaptive anti-tumor immune response was evidenced by the slightly decreased percentage of CD4+ helper cells among CD3+ lymphocytes, both in the blood and in the pancreas, but concurrent with a statistically significant increase in the percentage of CD8+ cells in the pancreas. Changes in the CD4+/CD8+ ratio suggest an enhanced immune response triggered by ADSC-assisted oncolytic therapy. Analysis of H&E-stained tissue specimens from all treatment groups showed that a five-dose therapy appears to be safe for the animals as no pathology was revealed in the livers. Cancer cell infiltrations in the pancreas and spleen were evidenced in the PBS^−^ control groups, whereas high lymphocyte infiltrates were present in specimens from the group treated with the ADSC-shielded virus; and minimal lymphocyte infiltrates were present in specimens from virus-treated group. The virus-treated groups revealed fibrotic strands suggestive of the eradication of cancer cells, further supported by the decreased mitotic index. Taken together, the results of flow cytometry and microscopic analysis suggest that vMyx-mLIGHT-Fluc/tdTr delivered by ADSCs was able to modulate the immune microenviroment in PDAC tissues and contribute to the prolonged survival of mice treated with the five-dose strategy.

We show in this proof-of concept study that the recombinant MYXV used, a therapeutic agent with a dual mode of action (as a tumor oncolytic agent and an elicitor of an acquired immune response) can be an efficient component of a multi-pronged approach to PDAC therapy. ADSC-mediated IP delivery of MYXV to treat orthotopic experimental PDAC lesions in mice proved to be highly effective. Our results demonstrated increased survival of animals bearing orthotopic PDAC tumor lesions following monotherapy with an engineered recombinant MYXV delivered by ADSC; they also showed increased numbers of T cells in pancreata dissected from the treated mice. This enables a more advanced approach to virus- and ADSC-based therapies.

The levels of productive MYXV replication in two human pancreatic cancer cell lines tested (AsPC-1 and Panc-1) showed lower susceptibility to infection yet marked-to-significant reductions in cell viability. This may obviously be a disadvantage in clinical oncology. Combinatory therapeutic approaches involving more advanced armed OVs triggering enhanced adaptive immunity effects, as well as agents targeting desmoplasia, should offset this drawback. Single-agent approaches seem insufficient to improve the therapeutic outcomes of PDAC [41].

Intelligent combinatorial therapies appear to be required to achieve significant synergism in PDAC treatment. For example, modern radiation techniques, eliciting abscopal effects via reconditioning of the tumor microenvironment, reprogramming tumor-infiltrating macrophages towards an M1-like phenotype and favoring the recruitment of adoptively transferred T cells [42] or activating cytosolic DNA sensors, such as STING, are on the horizon [43]. Another example are early phase clinical trials combining recombinant human hyaluronidase, gemcitabine and nab-paclitaxel, which have revealed promising results, particularly in those patients whose tumors were characterized by high levels of hyaluronan [44]. The rationale for all these approaches is to outcompete therapy resistance; this might be challenging, however, as combined modality treatments are frequently associated with higher toxicity levels [45]. Immunotherapy is another hope for novel strategies against pancreatic cancer, even though this deadly cancer has been so far resistant to immune checkpoint blockade and chimeric antigen receptor (CAR) T-cell therapies. Research involving the use of CAR T cells in pancreatic cancer therapy is rapidly developing, in combination with other treatments as well [46].

Overall, the successful strategies against PDAC should thus involve combinations of modern “classical” treatments with different immunotherapeutic approaches and, hopefully, with oncovirotherapy.

## 5. Conclusions

To sum up, we have shown that oncolytic monotherapy with adipose-derived mesenchymal stem cells loaded with LIGHT-expressing MYXV delivered IP to experimental orthotopic PDAC lesion-bearing mice resulted in increased animal survival and boosted the immune response.

## Data Availability

Data sharing not applicable.

## Supplementary Materials

The following are available online at https://www.mdpi.com/2072-6694/13/6/1394/s1, Figure S1: Schematic design showing the organization and recombination region of vMyx-mLIGHT-Fluc/tdTr, Figure S2: Constitutive expression of Light in infected and non-infected ADSCs, Figure S3: Expression and localization of murine LIGHT in the vMyx-mLIGHT-FLuc/tdTr-infected cells, Figure S4: Effect of intraperitoneally vs. intravenously injected MYXV construct (vMyx-mLIGHT-Fluc/tdTr) on biodistribution in mice, Figure S5: Histological appearance of representative H&E-stained tissue sections, Table S1: The source of adipose tissue-derived mesenchymal stem cells.

## References

[1] Rhim A.D., Mirek E.T., Aiello N.M., Maitra A., Bailey J.M., McAllister F., Reichert M., Beatty G.L., Rustgi A.K., Vonderheide R.H. (2012). EMT and dissemination precede pancreatic tumor formation. Cell.

[2] Dougan S.K. (2017). The Pancreatic Cancer Microenvironment. Cancer J..

[3] Erkan M., Kurtoglu M., Kleeff J. (2016). The role of hypoxia in pancreatic cancer: A potential therapeutic target?. Expert. Rev. Gastroenterol. Hepatol..

[4] Heinemann V., Reni M., Ychou M., Richel D.J., Macarulla T., Ducreux M. (2014). Tumour-stroma interactions in pancreatic ductal adenocarcinoma: Rationale and current evidence for new therapeutic strategies. Cancer Treat. Rev..

[5] Li N., Li Y., Li Z., Huang C., Yang Y., Lang M., Cao J., Jiang W., Xu Y., Dong J. (2016). Hypoxia Inducible Factor 1 (HIF-1) Recruits Macrophage to Activate Pancreatic Stellate Cells in Pancreatic Ductal Adenocarcinoma. Int. J. Mol. Sci..

[6] Ene-Obong A., Clear A.J., Watt J., Wang J., Fatah R., Riches J.C., Marshall J.F., Chin-Aleong J., Chelala C., Gribben J.G. (2013). Activated pancreatic stellate cells sequester CD8+ T cells to reduce their infiltration of the juxtatumoral compartment of pancreatic ductal adenocarcinoma. Gastroenterology.

[7] Ozdemir B.C., Pentcheva-Hoang T., Carstens J.L., Zheng X., Wu C.C., Simpson T.R., Laklai H., Sugimoto H., Kahlert C., Novitskiy S.V. (2014). Depletion of carcinomaassociated fibroblasts and fibrosis induces immunosuppression and accelerates pancreas cancer with reduced survival. Cancer Cell.

[8] Daniel S.K., Sullivan K.M., Labadie K.P., Pillarisetty V.G. (2019). Hypoxia as a barrier to immunotherapy in pancreatic adenocarcinoma. Clin. Transl. Med..

[9] Mitchem J.B., Brennan D.J., Knolhoff B.L., Belt B.A., Zhu Y., Sanford D.E., Belaygorod L., Carpenter D., Collins L., Piwnica-Worms D. (2013). Targeting tumor-infiltrating macrophages decreases tumor-initiating cells, relieves immunosuppression, and improves chemotherapeutic responses. Cancer Res..

[10] Hu H., Hang J.J., Han T., Zhuo M., Jiao F., Wang L.W. (2016). The M2 phenotype of tumor-associated macrophages in the stroma confers a poor prognosis in pancreatic cancer. Tumour Biol..

[11] Adamska A., Domenichini A., Falasca M. (2017). Pancreatic Ductal Adenocarcinoma: Current and Evolving Therapies. Int. J. Mol. Sci..

[12] Shah A.N., Summy J.M., Zhang J., Park S.I., Parikh N.U., Gallick G.E. (2007). Development and characterization of gemcitabine-resistant pancreatic tumor cells. Ann. Surg. Oncol..

[13] Wang Z., Li Y., Kong D., Banerjee S., Ahmad A., Azmi A.S., Ali S., Abbruzzese J.L., Gallick G.E., Sarkar F.H. (2009). Acquisition of epithelial-mesenchymal transition phenotype of gemcitabine-resistant pancreatic cancer cells is linked with activation of the notch signaling pathway. Cancer Res..

[14] Yang A.D., Camp E.R., Fan F., Shen L., Gray M.J., Liu W., Somcio R., Bauer T.W., Wu Y., Hicklin D.J. (2006). Vascular endothelial growth factor receptor-1 activation mediates epithelial to mesenchymal transition in human pancreatic carcinoma cells. Cancer Res..

[15] Vacchelli E., Eggermont A., Sautès-Fridman C., Galon J., Zitvogel L., Kroemer G., Galluzzi L. (2013). Trial watch: Oncolytic viruses for cancer therapy. Oncoimmunology.

[16] Zeyaullah M., Patro M., Ahmad I., Ibraheem K., Sultan P., Nehal M., Ali A. (2012). Oncolytic viruses in the treatment of cancer: A review of current strategies. Pathol. Oncol. Res..

[17] Torres-Domínguez L.E., de Matos A.L., Rahman M.M., McFadden G. (2021). Methods for the Construction of Recombinant Oncolytic Myxoma Viruses. Methods Mol. Biol..

[18] Pasero C., Barbarat B., Just-Landi S., Bernard A., Aurran-Schleinitz T., Rey J., Eldering E., Truneh A., Costello R.T., Olive D. (2009). A role for HVEM, but not lymphotoxin-beta receptor, in LIGHT-induced tumor cell death and chemokine production. Eur. J. Immunol..

[19] Maker A.V. (2016). Precise identification of immunotherapeutic targets for solid malignancies using clues within the tumor microenvironment-evidence to turn on the LIGHT. Oncoimmunology.

[20] Šedý J.R., Ramezani-Rad P. (2019). HVEM network signaling in cancer. Adv. Cancer Res..

[21] Dai S., Lv Y., Xu W., Yang Y., Liu C., Dong X., Zhang H., Prabhakar B.S., Maker A.V., Seth P. (2020). Oncolytic adenovirus encoding LIGHT (TNFSF14) inhibits tumor growth via activating anti-tumor immune responses in 4T1 mouse mammary tumor model in immune competent syngeneic mice. Cancer Gene Ther..

[22] Hadryś A., Sochanik A., McFadden G., Jazowiecka-Rakus J. (2020). Mesenchymal stem cells as carriers for systemic delivery of oncolytic viruses. Eur. J. Pharmacol..

[23] Jazowiecka-Rakus J., Sochanik A., Rusin A., Hadryś A., Fidyk W., Villa N., Rahman M.M., Chmielik E., Franco L.S., McFadden G. (2020). Myxoma Virus-Loaded Mesenchymal Stem Cells in Experimental Oncolytic Therapy of Murine Pulmonary Melanoma. Mol. Ther. Oncol..

[24] Kulu Y., Dorfman J.D., Kuruppu D., Fuchs B.C., Goodwin J.M., Fujii T., Kuroda T., Lanuti M., Tanabe K.K. (2009). Comparison of intravenous versus intraperitoneal administration of oncolytic herpes simplex virus 1 for peritoneal carcinomatosis in mice. Cancer Gene Ther..

[25] Wennier S.T., Liu J., Li S., Rahman M.M., Mona M., McFadden G. (2012). Myxoma virus sensitizes cancer cells to gemcitabine and is an effective oncolytic virotherapeutic in models of disseminated pancreatic cancer. Mol. Ther..

[26] Bartee E., Mohamed M.R., Lopez M.C., Baker H.V., McFadden G. (2009). The addition of tumor necrosis factor plus beta interferon induces a novel synergistic antiviral state against poxviruses in primary human fibroblasts. J. Virol..

[27] Zemp F.J., Lun X., McKenzie B.A., Zhou H., Maxwell L., Sun B., Kelly J.J., Stechishin O., Luchman A., Weiss S. (2013). Treating brain tumor-initiating cells using a combination of myxoma virus and rapamycin. Neuro. Oncol..

[28] Matsuda Y., Yoshimura H., Ishiwata T., Sumiyoshi H., Matsushita A., Nakamura Y., Aida J., Uchida E., Takubo K., Arai T. (2016). Mitotic index and multipolar mitosis in routine histologic sections as prognostic markers of pancreatic cancers: A clinicopathological study. Pancreatology.

[29] Rahman M.M., Bagdassarian E., Ali M.A.M., McFadden G. (2017). Identification of host DEAD-box RNA helicases that regulate cellular tropism of oncolytic Myxoma virus in human cancer cells. Sci. Rep..

[30] Amrutkar M., Gladhaug I.P. (2017). Pancreatic Cancer Chemoresistance to Gemcitabine. Cancers.

[31] Grasso C., Jansen G., Giovannetti E. (2017). Drug resistance in pancreatic cancer: Impact of altered energy metabolism. Crit. Rev. Oncol. Hematol..

[32] Morrison A.H., Byrne K.T., Vonderheide R.H. (2018). Immunotherapy and Prevention of Pancreatic Cancer. Trends Cancer.

[33] Draganov D.D., Santidrian A.F., Minev I., Nguyen D., Kilinc M.O., Petrov I., Vyalkova A., Lander E., Berman M., Minev B. (2019). Delivery of oncolytic vaccinia virus by matched allogeneic stem cells overcomes critical innate and adaptive immune barriers. J. Transl. Med..

[34] Shoyaib A.A., Archie S.R., Karamyan W.T. (2019). Intraperitoneal Route of Drug Administration: Should it Be Used in Experimental Animal Studies. Pharm. Res..

[35] Gujar S., Pol J.G., Kim Y., Lee P.W. (2018). Kroemer, G. Antitumor Benefits of Antiviral Immunity: An Underappreciated Aspect of Oncolytic Virotherapies. Trends Immunol..

[36] Stromnes I.M., Hulbert A., Pierce R.H., Greenberg P.D., Hingorani S.R. (2017). T-cell Localization, Activation, and Clonal Expansion in Human Pancreatic Ductal Adenocarcinoma. Cancer Immunol. Res..

[37] Poschke I., Faryna M., Bergmann F., Flossdorf M., Lauenstein C., Hermes J., Hinz U., Hank T., Ehrenberg R., Volkmar M. (2016). Identification of a tumor-reactive T-cell repertoire in the immune infiltrate of patients with resectable pancreatic ductal adenocarcinoma. Oncoimmunology.

[38] Connor A.A., Denroche R.E., Jang G.H., Timms L., Kalimuthu S.N., Selander I., McPherson T., Wilson G.W., Chan-Seng-Yue M.A., Borozan I. (2017). Association of Distinct Mutational Signatures with Correlates of Increased Immune Activity in Pancreatic Ductal Adenocarcinoma. JAMA Oncol..

[39] Balachandran V.P., Luksza M., Zhao J.N., Makarov V., Moral J.A., Remark R., Herbst B., Askan G., Bhanot U., Senbabaoglu Y. (2017). Identification of unique neoantigen qualities in long-term survivors of pancreatic cancer. Nature.

[40] Werner J., Combs S.E., Springfeld C., Hartwig W., Hackert T., Buchler M.W. (2013). Advanced-stage pancreatic cancer: Therapy options. Nat. Rev. Clin. Oncol..

[41] Orth M., Metzger P., Gerum S., Mayerle J., Schneider G., Belka C., Schnurr M., Lauber K. (2019). Pancreatic ductal adenocarcinoma: Biological hallmarks, current status, and future perspectives of combined modality treatment approaches. Radiat. Oncol..

[42] Klug F., Prakash H., Huber P.E., Seibel T., Bender N., Halama N., Pfirschke C., Voss R.H., Timke C., Umansky L. (2013). Low-dose irradiation programs macrophage differentiation to an iNOS(+)/M1 phenotype that orchestrates effective T cell immunotherapy. Cancer Cell.

[43] Baird J.R., Friedman D., Cottam B., Dubensky T.W., Kanne D.B., Bambina S., Bahjat K., Crittenden M.R., Gough M.J. (2016). Radiotherapy Combined with Novel STING-Targeting Oligonucleotides Results in Regression of Established Tumors. Cancer Res..

[44] Hingorani S.R., Zheng L., Bullock A.J., Seery T.E., Harris W.P., Sigal D.S., Braiteh F., Ritch P.S., Zalupski M.M., Bahary N. (2018). HALO 202: Randomized Phase II Study of PEGPH20 Plus Nab-Paclitaxel/Gemcitabine Versus Nab-Paclitaxel/ Gemcitabine in Patients with Untreated, Metastatic Pancreatic Ductal Adenocarcinoma. J. Clin. Oncol..

[45] Niyazi M., Maihoefer C., Krause M., Rodel C., Budach W., Belka C. (2011). Radiotherapy and “new” drugs-new side effects?. Radiat. Oncol..

[46] Ali A.I., Oliver A.J., Samiei T., Chan J.D., Kershaw M.H., Slaney C.Y. (2019). Genetic Redirection of T Cells for the Treatment of Pancreatic Cancer. Front. Oncol..

