# Supplementary material for: Myxoma Virus Expressing LIGHT (TNFSF14) Pre-Loaded into Adipose-Derived Mesenchymal Stem Cells Is Effective Treatment for Murine Pancreatic Adenocarcinoma
Source: Cancers (Basel). 2021 Mar 19;13(6):1394. doi: 10.3390/cancers13061394 (PMC8003548; doi:10.3390/cancers13061394)
Supplement: Supplementary file 1 [file cancers-13-01394-s001.pdf]

cancers MDPI

Supplementary Materials: Myxoma virus expressing LIGHT (TNFSF14) pre-loaded into adipose-derived mesenchymal stem cells is effective treatment for murine pancreatic adenocarcinoma

Joanna Jazowiecka-Rakus, Agata Hadrys, Masmudur M. Rahman, Grant McFadden, Wojciech Fidyk, Ewa Chmielik, Marlena Pazdzior, Maciej Grajek, Violetta Kozik, Aleksander Sochanik

Supplementary Information

1. ***Mus musculus*** **tumor necrosis factor (ligand) superfamily, member 14 (Tnfsf14) cDNA (NCBI Reference Sequence: NM_019418.3)**

ATGGAGAGTGTGGTACAGCCTTCAGTGTTTGTGGTGGATGGACAGACGGACATCCCATTCAGGCGGCTGGAA
CAGAACCACCGGAGACGGCGCTGTGGCACTGTCCAGGTCAGCCTGGCCCTGGTGCTGCTGCTAGGTGCTGGG
CTGGCCACTCAGGGCTGGTTTCTCCTGAGACTGCATCAACGTCTTGGAGACATAGTAGCTCATCTGCCAGATG
GAGGCAAAGGCTCCTGGGAGAAGCTGATACAAGATCAACGATCTCACCAGGCCAACCCAGCAGCACATCTT
ACAGGAGCCAACGCCAGCTTGATAGGTATTGGTGGACCTCTGTTATGGGAGACACGACTTGGCCTGGCCTTCT
TGAGGGGCTTGACGTATCATGATGGGGCCCTGGTGACCATGGAGCCCGGTTACTACTATGTGTACTCCAAAGT
GCAGCTGAGCGGCGTGGGCTGCCCCCAGGGGCTGGCCAATGGCCTCCCCATCACCCATGGACTATACAAGCG
CACATCCCGCTACCCGAAGGAGTTAGAACTGCTGGTCAGTCGGCGGTCACCCTGTGGCCGGGCCAACAGCTC
CCGAGTCTGGTGGGACAGCAGCTTCCTGGGCGGCGTGGTACATCTGGAGGCTGGGGAAGAGGTGGTGGTCCG
CGTGCCTGGAAACCGCCTGGTCAGACCACGTGACGGCACCAGGTCCTATTTCGGAGCTTTCATGGTCTGA

2. vMyx-mLIGHT-Fluc/tdTr Recombinant Virus

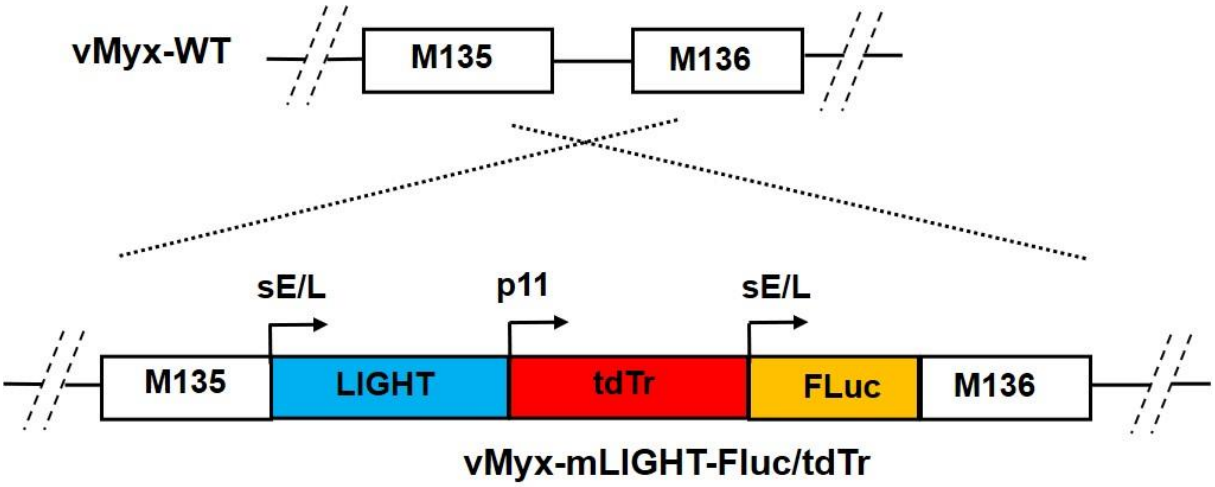

Figure S1. Schematic design showing the organization and recombination region of vMyx-mLIGHT-Fluc/tdTr. This recombinant MYXV expresses murine LIGHT under a poxvirus synthetic early/late promoter (sE/L), tdTomato red (tdTr) under a poxvirus late p11 promoter, and firefly luciferase (Fluc) under a sE/L promoter, all inserted between M135 and M136 genes.

3. RNA Isolation, cDNA Synthesis and RT-qPCR

Total RNA was isolated from infected (with vMyx-mLIGHT-Fluc/tdTr or vMyx-WT; MOI = 5) and non-infected ADSC cultures using RNeasy Mini Kit (Qiagen) according to the manufacturer's instructions. Synthesis of cDNA was performed from 250 ng of total RNA using the High-Capacity cDNA Reverse Transcription Kit (Applied Biosystems) according to the manufacturer's instructions. RT-qPCR reactions were performed in duplicate for each sample using a reaction mix prepared as follows: 1×SYBR Select Master Mix (Applied Biosystems), 2 μL of primers (forward 5′-GGAGACATAGTAGCTCATCTGCC-3′ and reverse 5′-CCACCAATACCTATCAAGCTGGC-3′; 1 μM each), and 4.0 μL of 20× diluted cDNA in a final volume of 15 μL. The amplification protocol included an initial preheating at 50 °C for 2 min, initial denaturation at 95 °C for 2 min, and 40 cycles of amplification (95 °C for 15 s and 60 °C for 60 s). Melting curve analyses were performed at the end of each run. RT-qPCR was carried out with a Rotor-Gene Q (Qiagen, Hilden, Germany). Constitutive expression of *Light* was rendered as a ratio of target gene (*Light*) *vs*. reference gene (glyceraldehyde 3-phosphate dehydrogenase - *GAPDH*) using the Pfaffl method [1].

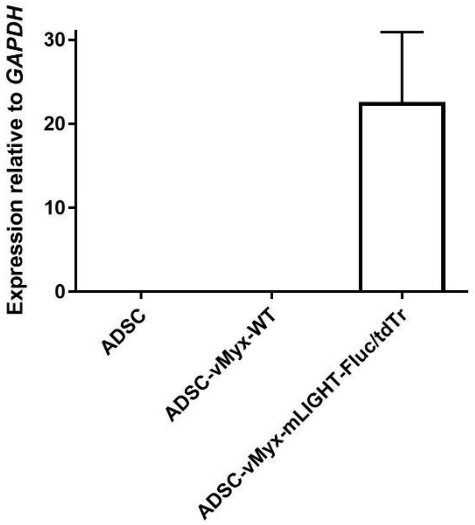

Figure S2. Constitutive expression of *Light* in infected and non-infected ADSCs. The gene transcript was measured using RT-qPCR in non-infected ADSCs or ADSCs infected with either vMyx-mLIGHT-Fluc/tdTr or with vMyx-WT. The data show mean ± SD of two independent experiments.

4. Immunofluorescence

HeLa cells (5 × 10^5/dish) were seeded on the bottom of 35-mm glass Petri dishes. Next day cells were infected with vMyx-mLIGHT-FLuc/tdTr (MOI = 3). After 24 h post infection, cells were washed three times with PBS^-, fixed with 2% paraformaldehyde in PBS^- (12 min/RT), washed again three times with PBS^- and permeabilized with Triton X-100 in PBS^- (0.1% / 90 sec /RT). Cells were then washed three times with PBS^- and blocked with 3% BSA in PBS^- (30 min/37 °C). Next, cells were incubated with primary rabbit polyclonal antibody (1:300 dilution) against LIGHT (Santa Cruz Biotechnology, FL-240) for 30 min at 37 °C. Cells were then washed six times with PBS^- and incubated with secondary antibody conjugated to Alexa Fluor 488 Goat anti-rabbit (Thermo Fisher Scientific). Finally, cells were washed six times with PBS^- and mounted on glass slides with VECTASHIELD® (Vectorlabs) containing DAPI (4′,6-diamidino-2-phenyl-indole) to stain nuclear DNA and viral factory. Micrographs were taken using a Leica fluorescence microscope.

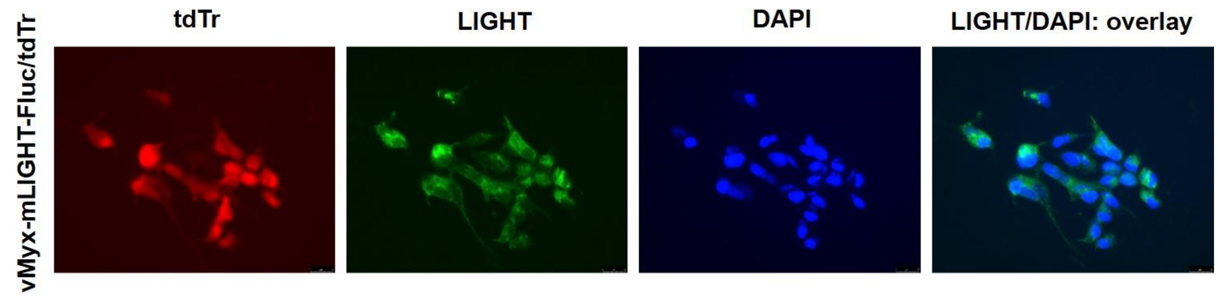

Figure S3. Expression and localization of murine LIGHT in cells infected with vMyx-mLIGHT-FLuc/tdTr. HeLa cells were infected with vMyx-mLIGHT-Fluc/tdTr (MOI = 3). After 24 h p.i., cells were fixed and stained with antibody against LIGHT. Nuclei were stained with DAPI. Micrographs were taken using a Leica fluorescence microscope.

5. Comparison of Intravenous *vs.* Intraperitoneal Delivery of ADSC-Shielded MYXV or Unshielded MYXV

C57BL/6 mice (*n* = 3) were orthotopically injected (day 0) with 1 × 10^6 Pan02 cells suspended in 30 µL PBS^-. Seven days after implantation, mice were intraperitoneally injected with a single dose of ADSCs infected for 24 h (MOI = 5) with vMyx-mLIGHT-Fluc/tdTr (5 × 10^5 cells suspended in 100 µL PBS^-) or with unshielded vMyx-mLIGHT-Fluc/tdTr (5 × 10^5 FFU/100 µL PBS^-). Bioluminescence imaging (BLI) of luciferase reporter gene expression was performed using Lumina IVIS Imaging System (PerkinElmer). At the 3-h time point p.i. mice were intraperitoneally injected with 1.5 mg D-luciferin (Promega) and bioluminescence images were acquired. Measurements in intact animals were immediately followed by examination of dissected organs (pancreas, spleen, liver, lungs, heart, kidneys, stomach and duodenum).

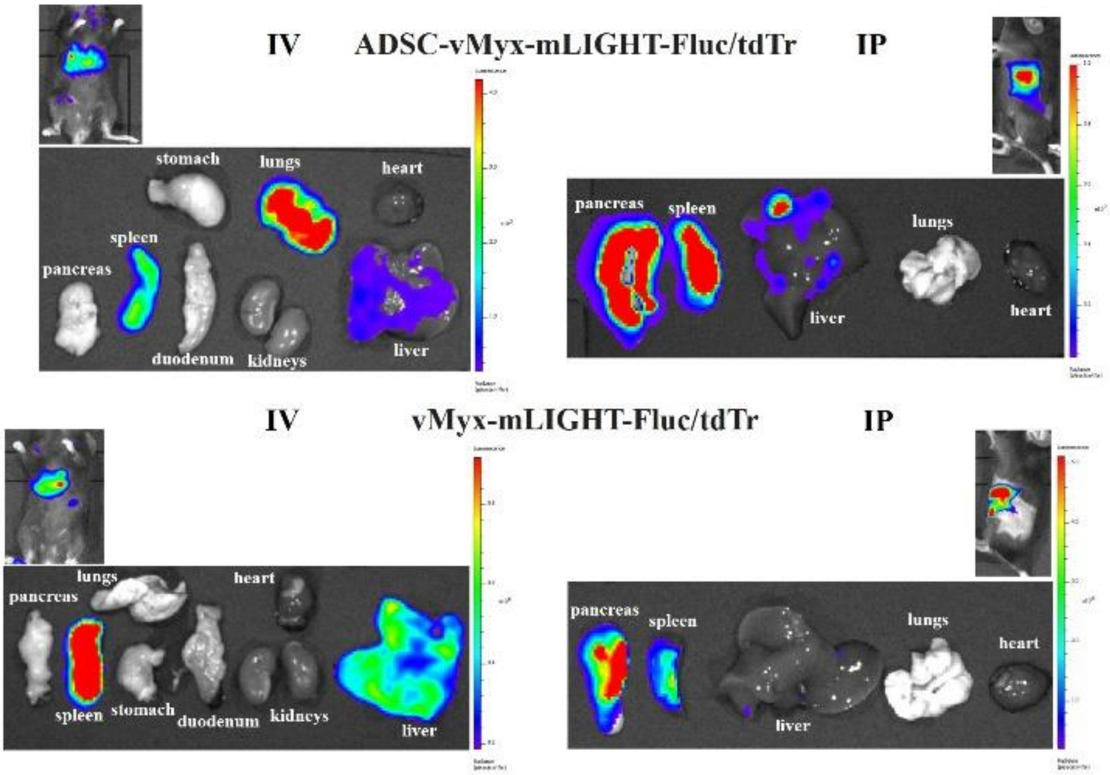

Figure S4. Effect of intraperitoneal *vs.* intravenous injection of MYXV construct (vMyx-mLIGHT-Fluc/tdTr) on biodistribution in mice. Bioluminescence (BLI) images were acquired in orthotopic pancreatic adenocarcinoma-bearing mice (+Pan02) at 3-h timepoint post injection (either IV or IP) of unshielded or ADSC-shielded MYXV construct (vMyx-mLIGHT-Fluc/tdTr); intact mice and dissected organs (pancreas, spleen, liver, lungs, heart, kidneys, stomach and duodenum) were examined. BLI expressed as radiance (photons/sec/cm^2/sr). Different radiance scales shown to cover the whole span of bioluminescence (*n* = 3/group).

6. Histological appearance of representative H&E-stained tissue sections

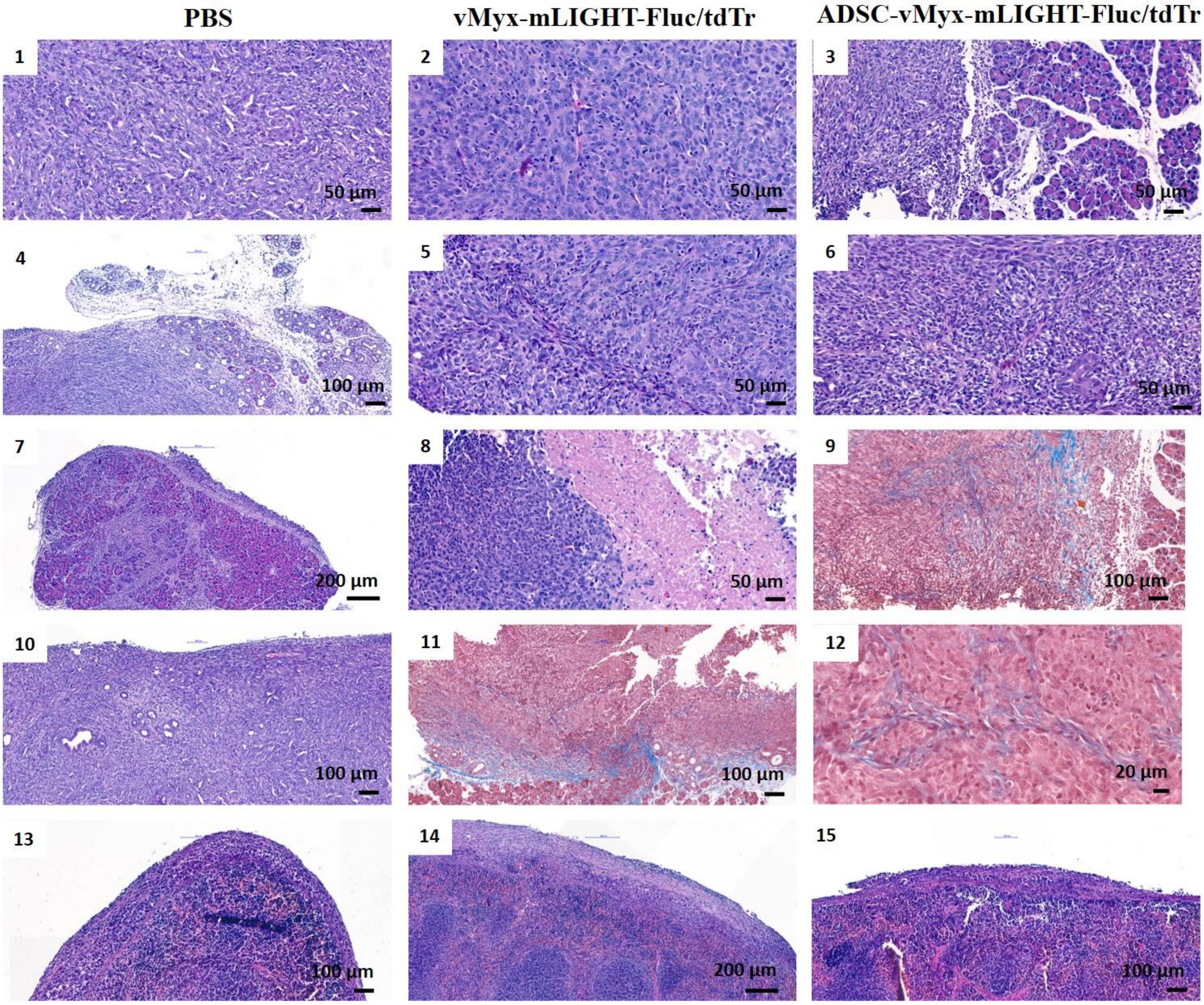

Figure S5. Histological appearance of representative H&E-stained tissue sections. Sections were obtained from the material collected on the 21st day of therapeutic experiment using LIGHT-expressing MYXV construct, either ADSC-shielded or unshielded (see Results; Figure 6). /1-2/ atypical mitoses; /3-4/ lymphocytic infiltrates; /5/ cells with hyperchromatic nucleus; /6/ tumor with less cellularity; /7/ tumor invading acinar cells; /8/ tumor necrosis; /9/ growth of connective tissue strands with reduced cellularity; /10/ Tumor surrounding interlobular ducts; /11/ connective tissue strands at the tumor periphery (Masson's staining); /12/ connective tissue strands with a stream pattern located next to cells (Masson's staining); /13-15/ infiltration of the spleen capsule; scale bars: 20-200 µm.

7. The source of adipose tissue-derived mesenchymal stem cells.

Table S1. The source of adipose tissue-derived mesenchymal stem cells.

Adipose Tissue Donors	Sex	Age	Reason for Surgery
Donor #1	male	40	Trauma-related reconstructive surgery *; free soft tissue flaps, abdominal area
Donor #2	female	35	Trauma-related reconstructive surgery *; free soft tissue flaps, abdominal area
Donor #3	female	43	Trauma-related reconstructive surgery *; free soft tissue flaps, abdominal area

* donors were not subjected to chemotherapy or radiotherapy.

Reference

1. Pfaffl, M.W. A new mathematical model for relative quantification in real-time RT–PCR. *Nucleic Acids Res* **2001**, *29*, e45.
